# Bilevel Parameter Learning for Higher-Order Total Variation Regularisation Models

**DOI:** 10.1007/s10851-016-0662-8

**Published:** 2016-06-01

**Authors:** J. C. De los Reyes, C.-B. Schönlieb, T. Valkonen

**Affiliations:** 1 Research Center on Mathematical Modelling (MODEMAT), Escuela Politécnica Nacional, Quito, Ecuador; 2 Department of Applied Mathematics and Theoretical Physics, University of Cambridge, Cambridge, UK; 3 Department of Mathematical Sciences, University of Liverpool, Liverpool, UK

**Keywords:** Bilevel optimisation, Total variation regularisers, Image quality measures

## Abstract

We consider a bilevel optimisation approach for parameter learning in higher-order total variation image reconstruction models. Apart from the least squares cost functional, naturally used in bilevel learning, we propose and analyse an alternative cost based on a Huber-regularised TV seminorm. Differentiability properties of the solution operator are verified and a first-order optimality system is derived. Based on the adjoint information, a combined quasi-Newton/semismooth Newton algorithm is proposed for the numerical solution of the bilevel problems. Numerical experiments are carried out to show the suitability of our approach and the improved performance of the new cost functional. Thanks to the bilevel optimisation framework, also a detailed comparison between $\text {TGV}^2$ and $\text {ICTV}$ is carried out, showing the advantages and shortcomings of both regularisers, depending on the structure of the processed images and their noise level.

## Introduction

In this paper, we propose a bilevel optimisation approach for parameter learning in higher-order total variation regularisation models for image restoration. The reconstruction of an image from imperfect measurements is essential for all research which relies on the analysis and interpretation of image content. Mathematical image reconstruction approaches aim to maximise the information gain from acquired image data by intelligent modelling and mathematical analysis.

A variational image reconstruction model can be formalised as follows: Given data *f* which is related to an image (or to certain image information, e.g. a segmented or edge detected image) *u* through a generic forward operator (or function) *K*, the task is to retrieve *u* from *f*. In most realistic situations, this retrieval is complicated by the ill-posedness of *K* as well as random noise in *f*. A widely accepted method that approximates this ill-posed problem by a well-posed one and counteracts the noise is the method of Tikhonov regularisation. That is, an approximation to the true image is computed as a minimiser of

1.1 $$\begin{aligned} \alpha ~ R(u) + d(K(u),f), \end{aligned}$$

where *R* is a regularising energy that models a-priori knowledge about the image *u*, $d(\cdot , \cdot )$ is a suitable distance function that models the relation of the data *f* to the unknown *u*, and $\alpha >0$ is a parameter that balances our trust in the forward model against the need of regularisation. The parameter $\alpha$, in particular, depends on the amount of ill-posedness in the operator *K* and the amount (amplitude) of the noise present in *f*. A key issue in imaging inverse problems is the correct choice of $\alpha$, image priors (regularisation functionals *R*), fidelity terms *d* and (if applicable) the choice of what to measure (the linear or non-linear operator *K*). Depending on this choice, different reconstruction results are obtained.

While functional modelling (1.1) constitutes a mathematically rigorous and physical way of setting up the reconstruction of an image—providing reconstruction guarantees in terms of error and stability estimates—it is limited with respect to its adaptivity for real data. On the other hand, data-based modelling of reconstruction approaches is set up to produce results which are optimal with respect to the given data. However, in general, it neither offers insights into the structural properties of the model nor provides comprehensible reconstruction guarantees. Indeed, we believe that for the development of reliable, comprehensible and at the same time effective models (1.1), it is essential to aim for a unified approach that seeks tailor-made regularisation and data models by combining model- and data-based approaches.

To do so, we focus on a bilevel optimisation strategy for finding an optimal setup of variational regularisation models (1.1). That is, for a given training pair of noisy and original clean images $(f,f_0)$, respectively, we consider a learning problem of the form

1.2 $$\begin{aligned} \min _\alpha F(u^*)= & {} cost(u^*,f_0) \quad \text {subject to}\nonumber \\&u^*\in \mathop {{{\mathrm{arg\,min}}}}\limits _{u} \left\{ \alpha ~ R(u) + d(K(u),f)\right\} , \end{aligned}$$

where *F* is a generic cost functional that measures the fitness of $u^*$ to the training image $f_0$. The argument of the minimisation problem will depend on the specific setup (i.e. the degrees of freedom) in the constraint problem (1.1). In particular, we propose a bilevel optimisation approach for learning optimal parameters in higher-order total variation regularisation models for image reconstruction in which the arguments of the optimisation constitute parameters in front of the first- and higher-order regularisation terms.

Rather than working on the discrete problem, as is done in standard parameter learning and model optimisation methods, we optimise the regularisation models in infinite-dimensional function space. The resulting problems are difficult to treat due to the non-smooth structure of the lower level problem, which makes it impossible to verify standard constraint qualification conditions for Karush–Kuhn–Tucker (KKT) systems. Therefore, in order to obtain characterising first-order necessary optimality conditions, alternative analytical approaches have emerged, in particular regularisation techniques [4, 20, 28]. We consider such an approach here and study the related regularised problem in depth. In particular, we prove the Fréchet differentiability of the regularised solution operator, which enables to obtain an optimality condition for the problem under consideration and an adjoint state for the efficient numerical solution of the problem. The bilevel problems under consideration are related to the emerging field of generalised mathematical programmes with equilibrium constraints (MPEC) in function space. Let us remark that even for finite-dimensional problems, there are few recent references dealing with stationarity conditions and solution algorithms for this type of problems (see, e.g. [18, 30, 33, 34, 38]).

Let us give an account to the state of the art of bilevel optimisation for model learning. In machine learning, bilevel optimisation is well established. It is a semi-supervised learning method that optimally adapts itself to a given dataset of measurements and desirable solutions. In [15, 23, 43], for instance, the authors consider bilevel optimisation for finite-dimensional Markov random field models. In inverse problems, the optimal inversion and experimental acquisition setup is discussed in the context of optimal model design in works by Haber, Horesh and Tenorio [25, 26], as well as Ghattas et al. [3, 9]. Recently, parameter learning in the context of functional variational regularisation models (1.1) also entered the image processing community with works by the authors [10, 22], Kunisch, Pock and co-workers [14, 33], Chung et al. [16] and Hintermüller et al. [30].

Apart from the work of the authors [10, 22], all approaches so far are formulated and optimised in the discrete setting. Our subsequent modelling, analysis and optimisation will be carried out in function space rather than on a discretisation of (1.1). While digitally acquired image data are of course discrete, the aim of high-resolution image reconstruction and processing is always to compute an image that is close to the real (analogue, infinite dimensional) world. Hence, it makes sense to seek images which have certain properties in an infinite dimensional function space. That is, we aim for a processing method that accentuates and preserves qualitative properties in images independent of the resolution of the image itself [45]. Moreover, optimisation methods conceived in function space potentially result in numerical iterative schemes which are resolution and mesh independent upon discretisation [29].

**Fig. 1:**
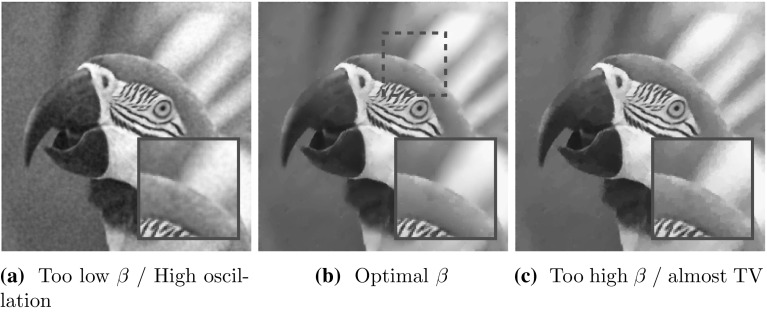
Effect of $\beta$ on $\text {TGV}^2$ denoising with optimal $\alpha$

Higher-order total variation regularisation has been introduced as an extension of the standard total variation regulariser in image processing. As the **T**otal **V**ariation (TV) [41] and many more contributions in the image processing community have proven, a non-smooth first-order regularisation procedure results in a non-linear smoothing of the image, smoothing more in homogeneous areas of the image domain and preserving characteristic structures such as edges. In particular, the TV regulariser is tuned towards the preservation of edges and performs very well if the reconstructed image is piecewise constant. The drawback of such a regularisation procedure becomes apparent as soon as images or signals (in 1D) are considered which do not only consist of constant regions and jumps but also possess more complicated, higher-order structures, e.g. piecewise linear parts. The artefact introduced by TV regularisation in this case is called staircasing [40]. One possibility to counteract such artefacts is the introduction of higher-order derivatives in the image regularisation. Chambolle and Lions [11], for instance, propose a higher-order method by means of an infimal convolution of the TV and the TV of the image gradient called **I**nfimal **C**onvolution **T**otal **V**ariation (ICTV) model. Other approaches to combine first- and second-order regularisation originate, for instance, from Chan et al. [12] who consider total variation minimisation together with weighted versions of the Laplacian, the Euler-elastica functional [13, 37], which combines total variation regularisation with curvature penalisation, and many more [35, 39] just to name a few. Recently, Bredies et al. have proposed **T**otal **G**eneralized **V**ariation (TGV) [5] as a higher-order variant of TV regularisation.

**Fig. 2:**
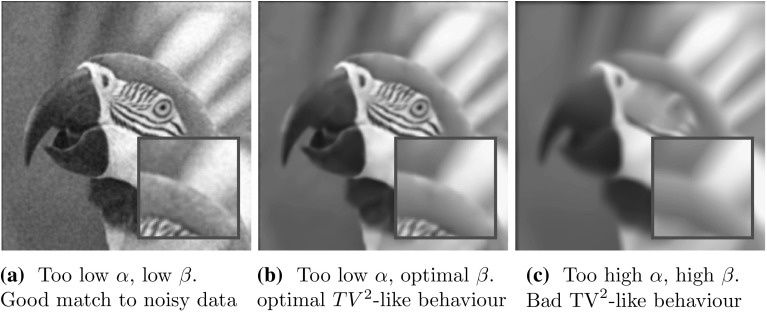
Effect of choosing $\alpha$ too large in $\text {TGV}^2$ denoising

In this work, we mainly concentrate on two second-order total variation models: the recently proposed TGV [5] and the ICTV model of Chambolle and Lions [11]. We focus on second-order TV regularisation only since this is the one which seems to be most relevant in imaging applications [6, 31]. For $\Omega \subset \mathbb R^2$ open and bounded and $u\in BV(\Omega )$, the ICTV regulariser reads

1.3 $$\begin{aligned} \text {ICTV}_{\alpha ,\beta }(u):= & {} \min _{v\in W^{1,1}(\Omega ),~ \nabla v\in BV(\Omega )} \alpha \Vert Du-\nabla v\Vert _{\mathcal {M}(\Omega ; \mathbb {R}^2)}\nonumber \\&+\, \beta \Vert D\nabla v\Vert _{\mathcal {M}(\Omega ; \mathbb {R}^{2\times 2})}. \end{aligned}$$

On the other hand, second-order TGV [7, 8] for $u\in BV(\Omega )$ reads

1.4 $$\begin{aligned} \text {TGV}^2_{\alpha ,\beta }(u):= & {} \min _{w \in BD(\Omega )} \alpha \Vert Du-w\Vert _{\mathcal {M}(\Omega ; \mathbb {R}^2)} \nonumber \\&+\, \beta \Vert Ew\Vert _{\mathcal {M}(\Omega ; {{\mathrm{Sym}}}^2(\mathbb {R}^2))}. \end{aligned}$$

Here

1.5 $$\begin{aligned} \Vert Du\Vert _{\mathcal {M}(\Omega ; \mathbb {R}^2)}= \sup _{\mathbf{g} \in C_0^\infty (\Omega ;\mathbb R^2), \Vert \mathbf g\Vert _\infty \le 1} \int _\Omega u ~\nabla \cdot \mathbf{g} ~\mathrm{d}x \end{aligned}$$

stands for the total variation of *u* in $\Omega$, $\text {BD}(\Omega ) :=\{ w \in L^1(\Omega ; \mathbb {R}^n) \mid \Vert Ew\Vert _{\mathcal {M}(\Omega ; \mathbb {R}^{n\times n})} < \infty \}$ is the space of vector fields of bounded deformation on $\Omega$, *E* denotes the *symmetrised gradient* and $\mathrm {Sym}^2(\mathbb {R}^2)$ denotes the space of symmetric tensors of order 2 with arguments in $\mathbb {R}^2$. The parameters $\alpha ,\beta$ are fixed positive parameters and will constitute the arguments in the special learning problem á la (1.2) we consider in this paper. The main difference between (1.3) and (1.4) is that we do not generally have that $w=\nabla v$ for any function *v*. That results in some qualitative differences of ICTV and TGV regularisation, compare for instance [1]. Substituting $\alpha R(u)$ in (1.1) by $\text {TGV}^2_{\alpha ,\beta }(u)$ or $\text {ICTV}_{\alpha ,\beta }(u)$ gives the TGV image reconstruction model and the ICTV image reconstruction model, respectively. In this paper, we only consider the case $K=Id$ identity and $d(u,f)=\Vert u-f\Vert _{L^2(\Omega )}^2$ in (1.1) which corresponds to an image denoising model for removing Gaussian noise. With our choice of regulariser, the former scalar $\alpha$ in (1.1) has been replaced by a vector $(\alpha ,\beta )$ of two parameters in (1.3) and (1.4). The choice of the entries in this vector now do not only determine the overall strength of the regularisation (depending on the properties of *K* and the noise level), but those parameters also balance between the different orders of regularity of the function *u*, and their choice is indeed crucial for the image reconstruction result. Large $\beta$ will give regularised solutions that are close to TV regularised reconstructions, compare Fig. 1. Large $\alpha$ will result in TV$^2$ type solutions, that is solutions that are regularised with TV of the gradient [27, 39], compare Fig. 2. With our approach described in the next section, we propose a learning approach for choosing those parameters optimally, in particular optimally for particular types of images.

For the existence analysis of an optimal solution as well as for the derivation of an optimality system for the corresponding learning problem (1.2), we will consider a smoothed version of the constraint problem (1.1)—which is the one in fact used in the numerics. That is, we replace *R*(*u*)—being TV, TGV or ICTV in this paper—by a Huber-regularised version and add an $H^1$ regularisation with a small weight to (1.1). In this setting and under the special assumption of box constraints on $\alpha$ and $\beta$, we provide a simple existence proof for an optimal solution. A more general existence result that holds also for the original non-smooth problem and does not require box constraints is derived in [19], and we refer the reader to this paper for a more sophisticated analysis on the structure of solutions.

A main challenge in the setup of such a learning approach is to decide what is the best way to measure fitness (optimality) of the model. In our setting this amounts to choosing an appropriate distance *F* in (1.2) that measures the fitness of reconstructed images to the ‘perfect’, noise-free images in an appropriate training set. We have to formalise what we mean by an optimal reconstruction model. Classically, the difference between the original, noise-free image $f_0$ and its regularised version $u_{\alpha ,\beta }$ is computed with an ${L_2^2}$ cost functional

1.6 $$\begin{aligned} F_{{L_2^2}}(u_{\alpha ,\beta }) := \Vert u_{\alpha ,\beta } - f_0\Vert _{L^2(\Omega )}^2, \end{aligned}$$

which is closely related to the PSNR quality measure. Apart from this, we propose in this paper an alternative cost functional based on a Huberised total variation cost

1.7 $$\begin{aligned} F_{{L_\eta ^1\!\nabla }}(u_{\alpha ,\beta }) :=\int _\Omega |D(u_{\alpha ,\beta }-f_0)|_{\gamma }~ dx, \end{aligned}$$

where the Huber regularisation $|\cdot |_{\gamma }$ will be defined later on in Definition 2.1. We will see that the choice of this cost functional is indeed crucial for the qualitative properties of the reconstructed image.

The proposed bilevel approach has an important indirect consequence: It establishes a basis for the comparison of the different total variation regularisers employed in image denoising tasks. In the last part of this paper, we exhaustively compare the performance of $\text {TV}$, $\text {TGV}^2$ and $\text {ICTV}$ for various image datasets. The parameters are chosen optimally, according to the proposed bilevel approach, and different quality measures (like PSNR and SSIM) are considered for the comparison. The obtained results are enlightening about when to use each one of the considered regularisers. In particular, $\text {ICTV}$ appears to behave better for images with arbitrary structure and moderate noise levels, whereas $\text {TGV}^2$ behaves better for images with large smooth areas.

**Outline of the paper** In Sect. 2, we state the bilevel learning problem for the two higher-order total variation regularisation models, TGV and ICTV, and prove existence of an optimal parameter pair $\alpha ,\beta$. The bilevel optimisation problem is analysed in Sect. 3, where existence of Lagrange multipliers is proved and an optimality system, as well as a gradient formula, is derived. Based on the optimality condition, a BFGS algorithm for the bilevel learning problem is devised in Sect. 5.1. For the numerical solution of each denoising problem, an infeasible semismooth Newton method is considered. Finally, we discuss the performance of the parameter learning method by means of several examples for the denoising of natural photographs in Sect. 5. Therein, we also present a statistical analysis on how TV, ICTV and TGV regularisation compare in terms of returned image quality, carried out on 200 images from the Berkeley segmentation dataset BSDS300.

## Problem Statement and Existence Analysis

We strive to develop a parameter learning method for higher-order total variation regularisation models that maximises the fit of the reconstructed images to training images simulated for an application at hand. For a given noisy image $f\in L^2(\Omega )$, $\Omega \subset \mathbb R^2$ open and bounded, we consider

2.1 $$\begin{aligned} \min _u \left\{ R_{\alpha , \beta }(u) + \frac{1}{2} \Vert u-f\Vert _{L^2(\Omega )}^2\right\} . \end{aligned}$$

where, $\alpha ,\beta \in \mathbb R$. We focus on TGV$^2$,

$$\begin{aligned} R_{\alpha ,\beta }(u)= & {} \text {TGV}^2_{\alpha ,\beta }(u):=\min _{w \in BD(\Omega )} \Vert \alpha ~(Du-w)\Vert _{\mathcal {M}(\Omega ; \mathbb {R}^2)} \\&+\, \Vert \beta ~Ew\Vert _{\mathcal {M}(\Omega ; {{\mathrm{Sym}}}^2(\mathbb {R}^2))}, \end{aligned}$$

and ICTV,

$$\begin{aligned} R_{\alpha ,\beta }(u)= & {} \text {ICTV}_{\alpha , \beta }(u):=\min _{\begin{array}{c} v\in W^{1,1}(\Omega )\\ \nabla v\in BV(\Omega ) \end{array}} \Vert \alpha ~(Du{-}\nabla v)\Vert _{\mathcal {M}(\Omega ; \mathbb {R}^2)} \\&+\, \Vert \beta ~D\nabla v\Vert _{\mathcal {M}(\Omega ; \mathbb {R}^{2\times 2})}, \end{aligned}$$

for $u\in BV(\Omega )$. For these models, we want to determine the optimal choice of $\alpha ,\beta$, given a particular type of images and a fixed noise level. More precisely, we consider a training pair $(f,f_0)$, where *f* is a noisy image corrupted by normally distributed noise with a fixed variation, and the image $f_0$ represents the ground truth or an image that approximates the ground truth within a desirable tolerance. Then, we determine the optimal choice of $\alpha ,\beta$ by solving the following problem:

2.2 $$\begin{aligned} \min _{(\alpha ,\beta )\in \mathbb R^{2}} ~ F(u_{\alpha ,\beta }) \quad \text { s.t. } \alpha , \beta \ge 0, \end{aligned}$$

where $F$ equals the ${L_2^2}$ cost (1.6) or the Huberised TV cost (1.7) and $u_{\alpha ,\beta }$ for a given *f* solves a regularised version of the minimisation problem (2.1) that will be specified in the next section, compare problem (2.3b). This regularisation of the problem is a technical requirement for solving the bilevel problem that will be discussed in the sequel. In contrast to learning $\alpha ,\beta$ in (2.1) in finite dimensional parameter spaces (as is the case in machine learning), we consider optimisation techniques in infinite dimensional function spaces.

### Formal Statement

Let $\Omega \subset \mathbb {R}^n$ be an open bounded domain with Lipschitz boundary. This will be our image domain. Usually $\Omega =(0, w) \times (0, h)$ for *w* and *h* the width and height of a two-dimensional image, although no such assumptions are made in this work. Our data *f* and $f_0$ are assumed to lie in $L^2(\Omega )$.

In our learning problem, we look for parameters $(\alpha ,\beta )$ that for some cost functional $F: H^1(\Omega ) \rightarrow \mathbb {R}$ solve the problem

2.3a $$\begin{aligned} \min _{(\alpha ,\beta )\in \mathbb R^{2}} ~ F(u_{\alpha ,\beta }) \end{aligned}$$

subject to

2.3b $$\begin{aligned}&u_{\alpha ,\beta } \in \mathop {{{\mathrm{arg\,min}}}}\limits _{u\in H^1(\Omega )} J^{\gamma ,\mu }(u; \alpha ,\beta ) \end{aligned}$$

2.3c $$\begin{aligned}&\alpha , \beta \ge 0, \end{aligned}$$

where

$$\begin{aligned} J^{\gamma ,\mu }(u; \alpha ,\beta ) :=\frac{1}{2} \Vert u-f\Vert _{L^2(\Omega )}^2 + R_{\alpha ,\beta }^{\gamma ,\mu }(u). \end{aligned}$$

Here $J^{\gamma ,\mu }(\cdot ; \alpha ,\beta )$ is the regularised denoising functional that amends the regularisation term in (2.1) by a Huber-regularised version of it with parameter $\gamma >0$, and an elliptic regularisation term with parameter $\mu >0$. In the case of TGV$^2$, the modified regularisation term $R_{\alpha ,\beta }^{\gamma ,\mu }(u)$ then reads, for $u\in H^1(\Omega )$,

$$\begin{aligned} \text {TGV}^{2,\gamma ,\mu }_{\alpha ,\beta }(u)&:=\min _{w \in H^1(\Omega )} \int _\Omega \alpha ~|Du-w|_\gamma ~ \mathrm{d}x\\&\quad \, +\, \int _\Omega \beta ~|Ew|_\gamma ~ \mathrm{d}x\\&\quad \, + \,\frac{\mu }{2} \left( \Vert u\Vert _{H^1(\Omega )}^2 + \Vert w\Vert _{\mathbb H^1(\Omega )}^2\right) , \end{aligned}$$

and in the case of ICTV, we have

$$\begin{aligned} \text {ICTV}_{\alpha , \beta }^{\gamma ,\mu }(u)&:=\min _{\begin{array}{c} v\in W^{1,1}(\Omega )\\ \nabla v\in BV(\Omega ,\mathbb R^n)\cap \mathbb H^1(\Omega ) \end{array}} \int _\Omega \alpha ~|Du-\nabla v|_\gamma ~\mathrm{d}x \\&\,\quad +\, \int _\Omega \beta ~|D\nabla v|_\gamma ~ \mathrm{d}x\\&\quad \, +\, \frac{\mu }{2} \left( \Vert u\Vert _{H^1(\Omega )}^2 + \Vert \nabla v\Vert _{\mathbb H^1(\Omega )}^2\right) . \end{aligned}$$

Here, $\mathbb H^1(\Omega )=H^1(\Omega ;\mathbb R^n)$ and the Huber regularisation $|\cdot |_\gamma$ is defined as follows.

#### Definition 2.1

Given $\gamma \in (0, \infty ]$, we define for the norm $\Vert \,\varvec{\cdot }\,\Vert _2$ on $\mathbb {R}^m$, the Huber regularisation

$$\begin{aligned} |g|_{\gamma } = {\left\{ \begin{array}{ll} \Vert g\Vert _2 - \frac{1}{2\gamma }, &{} \Vert g\Vert _2 \ge 1/\gamma , \\ \frac{\gamma }{2}\Vert g\Vert _2^2, &{} \Vert g\Vert _2 < 1/\gamma , \end{array}\right. } \end{aligned}$$

and its derivative, given by

2.4 $$\begin{aligned} h_\gamma (g):= \frac{\gamma g}{\max (1,\gamma |g|)}. \end{aligned}$$

For the cost functional $F$, given noise-free data $f_0 \in L^2(\Omega )$ and a regularised solution $u\in H^1(\Omega )$, we consider in particular the $L^2$ cost

$$\begin{aligned} F_{{L_2^2}}(u) = \frac{1}{2}\Vert f_0 - u\Vert _{L^{2}(\Omega ; \mathbb {R}^{d})}^2, \end{aligned}$$

as well as the Huberised total variation cost

$$\begin{aligned} F_{{L_\eta ^1\!\nabla }}(u) = \int _\Omega |D(f_0-u)|_{\gamma }~ dx \end{aligned}$$

with noise-free data $f_0 \in \text {BV}(\Omega )$.

#### Remark 2.1

Please note that in our formulation of the bilevel problem (2.3), we only impose a non-negativity constraint on the parameters $\alpha$ and $\beta$, i.e. we do not strictly bound them away from zero. There are two reasons for that. First, for the existence analysis of the smoothed problem, the case $\alpha =\beta =0$ is not critical since compactness can be secured by the $H^1$ term in the functional, compare Sect. 2.2. Second, in [19], we indeed prove that even for the non-smooth problem (as $\mu \rightarrow 0$), under appropriate assumptions on the given data, the optimal $\alpha ,\beta$ are guaranteed to be strictly positive.

### Existence of an Optimal Solution

The existence of an optimal solution for the learning problem (2.3) is a special case of the class of bilevel problems considered in [19], where the existence of optimal parameters in $(0,+\infty ]^{2N}$ is proven. For convenience of the reader, we provide a simplified proof for the case where additional box constraints on the parameters are imposed. We start with an auxiliary lower semicontinuity result for the Huber-regularised functionals.

#### Lemma 2.1

Let $u,v\in L^p(\Omega )$, $1\le p<\infty$. Then, the functional $u \mapsto \int _\Omega |u-v|_\gamma ~ dx$, where $|\cdot |_\gamma$ is the Huber regularisation in Definition 2.1, is lower semicontinuous with respect to weak* convergence in $\mathcal {M}(\Omega ; \mathbb {R}^d)$

#### Proof

Recall that for $g \in \mathbb {R}^m$, the Huber-regularised norm may be written in dual form as

$$\begin{aligned} |g|_{\gamma }= \sup \Bigl \{ \langle q,g\rangle - \frac{\gamma }{2} \Vert q\Vert _2^2 : \Vert q\Vert _2 \le 1 \Bigr \}. \end{aligned}$$

Therefore, we find that

$$\begin{aligned} G(u):= & {} \int _\Omega |u-v|_\gamma ~ dx=\sup \Big \{ \int _\Omega u(x)\cdot \varphi (x)~ \mathrm{d}x\\&- \int _\Omega \frac{\gamma }{2} \Vert \varphi (x)\Vert _2^2 \,\mathrm{d} x : \\&\quad \varphi \in C_c^\infty (\Omega ),\ \Vert \varphi (x)\Vert _2 \le 1 \text { for every } x \in \Omega \Big \}. \end{aligned}$$

The functional *G* is of the form $G(u) = \sup \{\langle u,\varphi \rangle -G^*(\varphi )\}$, where $G^*$ is the convex conjugate of *G*. Now, let $\{u^i\}_{i=1}^\infty$ converge to *u* weakly* in $\mathcal {M}(\Omega ; \mathbb {R}^d)$. Taking a supremising sequence $\{\varphi ^j\}_{j=1}^\infty$ for this functional at any point *u*, we easily see lower semicontinuity by considering the sequences $\{\langle u^i,\varphi ^j\rangle -G^*(\varphi ^j)\}_{i=1}^\infty$ for each *j*.$\square$

Our main existence result is the following.

#### Theorem 2.1

We consider the learning problem (2.3) for TGV$^2$ and ICTV regularisation, optimising over parameters $(\alpha ,\beta )$ such that $0 \le \alpha \le \bar{\alpha }, 0 \le \beta \le \bar{\beta }$. Here $(\bar{\alpha },\bar{\beta })<\infty$ is an arbitrary but fixed vector in $\mathbb R^{2}$ that defines a box constraint on the parameter space. There exists an optimal solution $(\hat{\alpha },\hat{\beta })\in \mathbb R^{2}$ for this problem for both choices of cost functionals, $F=F_{L^2_2}$ and $F=F_{{L_\eta ^1\!\nabla }}$.

#### Proof

Let $(\alpha _n,\beta _n)\subset \mathbb R^{2}$ be a minimising sequence. Due to the box constraints we have that the sequence $(\alpha _n,\beta _n)$ is bounded in $\mathbb R^{2}$. Moreover, we get for the corresponding sequences of states $u_n:= u_{(\alpha _n,\beta _n)}$ that

$$\begin{aligned} J^{\gamma ,\mu }(u_n; \alpha _n,\beta _n) \le J^{\gamma ,\mu }(u; \alpha _n,\beta _n), \quad \forall u\in H^1(\Omega ), \end{aligned}$$

in particular this holds for $u=0$. Hence,

2.5 $$\begin{aligned} \frac{1}{2} \Vert u_n-f\Vert _{L^2(\Omega )}^2 + R_{\alpha _n,\beta _n}^{\gamma ,\mu }(u_n) \le \frac{1}{2} \Vert f\Vert _{L^2(\Omega )}^2. \end{aligned}$$

Exemplarily, we consider here the case for the TGV regulariser, that is $R_{\alpha _n,\beta _n}^{\gamma ,\mu } = \text {TGV}^{2,\gamma ,\mu }_{\alpha _n,\beta _n}$. The proof for the ICTV regulariser can be done in a similar fashion. Inequality (2.5) in particular gives

$$\begin{aligned} \Vert u_n\Vert _{H^1(\Omega )}^2 + \Vert w_n\Vert _{\mathbb H^1(\Omega )}^2 \le \frac{1}{\mu } \Vert f\Vert _{L^2(\Omega )}, \end{aligned}$$

where $w_n$ is the optimal *w* for $u_n$. This gives that $(u_n,w_n)$ is uniformly bounded in $H^1(\Omega )\times \mathbb H^1(\Omega )$ and that there exists a subsequence $\{(\alpha _n,\beta _n,u_n,w_n)\}$ which converges weakly in $\mathbb R^{2}\times H^1(\Omega )\times \mathbb H^1(\Omega )$ to a limit point $(\hat{\alpha },\hat{\beta },\hat{u},\hat{w})$. Moreover, $u_n\rightarrow \hat{u}$ strongly in $L^p(\Omega )$ and $w_n\rightarrow \hat{w}$ in $L^p(\Omega ;\mathbb R^n)$. Using the continuity of the $L^2$ fidelity term with respect to strong convergence in $L^2$, and the weak lower semicontinuity of the $H^1$ term with respect to weak convergence in $H^1$ and of the Huber-regularised functional even with respect to weak$*$ convergence in $\mathcal M$ (cf. Lemma 2.1), we get

$$\begin{aligned}&\frac{1}{2} \Vert \hat{u}-f\Vert _{L^2(\Omega )}^2 + \int _\Omega \hat{\alpha }~|D\hat{u}-\hat{w}|_\gamma ~ \mathrm{d}x + \int _\Omega \hat{\beta }~|Ew|_\gamma ~ \mathrm{d}x \\&\qquad +\, \frac{\mu }{2} \left( \Vert \hat{u}\Vert _{H^1(\Omega )}^2 + \Vert \hat{w}\Vert _{\mathbb H^1(\Omega )}^2\right) \\&\quad \le \liminf _n \frac{1}{2} \Vert u_n-f\Vert _{L^2(\Omega )}^2 \\&\qquad +\, \int _\Omega \hat{\alpha }~|Du_n-w_n|_\gamma ~ \mathrm{d}x + \int _\Omega \hat{\beta }~|Ew_n|_\gamma ~ \mathrm{d}x \\&\qquad + \frac{\mu }{2} \left( \Vert u_n\Vert _{H^1(\Omega )}^2 + \Vert w_n\Vert _{\mathbb H^1(\Omega )}^2\right) \\&\quad \le \liminf _n \frac{1}{2} \Vert u_n-f\Vert _{L^2(\Omega )}^2 + \int _\Omega \alpha _n~|Du_n-w_n|_\gamma ~ \mathrm{d}x \\&\qquad +\, \int _\Omega \beta _n~|Ew_n|_\gamma ~ \mathrm{d}x \\&\qquad +\, \frac{\mu }{2} \left( \Vert u_n\Vert _{H^1(\Omega )}^2 + \Vert w_n\Vert _{\mathbb H^1(\Omega )}^2\right) , \end{aligned}$$

where in the last step we have used the boundedness of the sequence $R_{\alpha _n,\beta _n}^{\gamma ,\mu }(u_n)$ from (2.5) and the convergence of $(\alpha _n,\beta _n)$ in $\mathbb R^{2}$. This shows that the limit point $\hat{u}$ is an optimal solution for $(\hat{\alpha },\hat{\beta })$. Moreover, due to the weak lower semicontinuity of the cost functional *F* and the fact that the set $\{(\alpha ,\beta ):~ 0 \le \alpha \le \bar{\alpha },0 \le \beta \le \bar{\beta }\}$ is closed, we have that $(\hat{\alpha },\hat{\beta },\hat{u})$ is optimal for (2.3). $\square$

#### Remark 2.2

- Using the existence result in [19], in principle we could allow infinite values for $\alpha$ and $\beta$. This would include both $\text {TV}^2$ and $\text {TV}$ as possible optimal regularisers in our learning problem.
- In [19], in the case of the $L^2$ cost and assuming that
  $$\begin{aligned} R_{\alpha ,\beta }^{\gamma }(f)>R_{\alpha ,\beta }^{\gamma }(f_0), \end{aligned}$$
  we moreover show that the parameters $(\alpha ,\beta )$ are strictly larger than 0. In the case of the Huberised TV cost, this is proven in a discretised setting. Please see [19] for details.
- The existence of solutions with $\mu =0$, that is without elliptic regularisation, is also proven in [19]. Note that here, we focus on the $\mu >0$ case since the elliptic regularity is required for proving the existence of Lagrange multipliers in the next section.

#### Remark 2.3

In [19], it was shown that the solution map of our bilevel problem is outer semicontinuous. This implies, in particular, that the minimisers of the regularised bilevel problems converge towards the minimiser of the original one.

## Lagrange Multipliers

In this section, we prove the existence of Lagrange multipliers for the learning problem (2.3) and derive an optimality system that characterises stationary points. Moreover, a gradient formula for the reduced cost functional is obtained, which plays an important role in the development of fast solution algorithms for the learning problems (see Sect. 5.1).

In what follows, all proofs are presented for the $\text {TGV}^2$ regularisation case, that is $R_{\alpha ,\beta }^{\gamma }=\text {TGV}^{2,\gamma }_{\alpha ,\beta }$. However, possible modifications to cope with the ICTV model will also be commented. Moreover, we consider along this section a smoother variant of the Huber regularisation, given by

$$\begin{aligned} |g|_\gamma = {\left\{ \begin{array}{ll} |g|+ \frac{\gamma }{2} L_\gamma -\frac{U_\gamma }{2} + \frac{A_\gamma }{\gamma ^2}+ \frac{B_\gamma }{\gamma ^3}+ \frac{C_\gamma }{3 \gamma ^4} \left( 3+ \frac{1}{4 \gamma ^2} \right) &{}\quad \text { if }~\gamma |g| \ge 1+ \frac{1}{2\gamma }\\ A_\gamma |g| +\frac{B_\gamma }{2} |g|^2+ \frac{C_\gamma }{3} |g|^3+D_\gamma &{}\quad \text { if }~1-\frac{1}{2\gamma }\le \gamma |g| \le 1+\frac{1}{2\gamma }\\ \frac{\gamma }{2} |g|^2 &{}\quad \text { if }~\gamma |g| \le 1-\frac{1}{2\gamma }, \end{array}\right. } \end{aligned}$$

with

$$\begin{aligned}&U_\gamma = \frac{1}{\gamma } \left( 1+ \frac{1}{2\gamma } \right) , \quad L_\gamma = \frac{1}{\gamma } \left( 1- \frac{1}{2\gamma } \right) ,\\&A_\gamma = 1- \frac{\gamma }{2} \left( \frac{2 \gamma +1}{2 \gamma } \right) ^2,\\&B_\gamma = \frac{\gamma }{2} (2 \gamma +1), \quad C_\gamma = - \frac{\gamma ^3}{2},\\&D_\gamma = - \frac{\gamma ^3}{3} L_\gamma ^3 - A_\gamma L_\gamma . \end{aligned}$$

This modified Huber function is required in order to get differentiability of the solution operator, a matter which is investigated next.

### Differentiability of the Solution Operator

We recall that the $\text {TGV}^2$ denoising problem can be rewritten as

$$\begin{aligned} y=(u,w)= & {} \mathop {{{\mathrm{arg\,min}}}}\limits _{BV(\Omega ) \times BD(\Omega )} \left\{ \frac{1}{2}\int _\Omega |u-f|^2 \right. \\&\left. + \int _\Omega \alpha |Du-w|_\gamma + \int _\Omega \beta |E w|_\gamma \right\} . \end{aligned}$$

Using an elliptic regularisation, we then get

$$\begin{aligned} y= & {} \mathop {{{\mathrm{arg\,min}}}}\limits _{H^1(\Omega ) \times \mathbb H^1(\Omega )} \left\{ \frac{1}{2} a(y,y)+ \frac{1}{2} \int _\Omega |u-f|^2 \right. \\&\left. + \int _\Omega \alpha |Du-w|_\gamma + \int _\Omega \beta |E w|_\gamma \right\} , \end{aligned}$$

where $a(y,y)= \mu \left( \Vert u\Vert _{H^1}^2 + \Vert w\Vert _{\mathbb H^1}^2 \right)$. A necessary and sufficient optimality condition for the latter is then given by the following variational equation:

3.1 $$\begin{aligned}&a(y, \Psi )+ \int _\Omega \alpha h_\gamma (Du-w)(D \phi - \varphi ) \,\mathrm{d}x\nonumber \\&\quad + \int _\Omega \beta h_\gamma (E w) E \varphi \,\mathrm{d}x +\int _\Omega (u-f)\phi \,dx=0,\nonumber \\&\quad \text { for all } \Psi \in Y, \end{aligned}$$

where $$\Psi =(\phi ,\varphi )$$, $$Y=H^1(\Omega ) \times \mathbb H^1(\Omega )$$ and

3.2 $$\begin{aligned} h_{\gamma }(g)= {\left\{ \begin{array}{ll} \frac{g}{|g|} &{}\;\text { if }~\gamma |g| \ge 1+ \frac{1}{2\gamma }\\ \frac{g}{|g|} (1- \frac{\gamma }{2} (1{-} \gamma |g|{+}\frac{1}{2\gamma })^2) &{}\;\text { if }~1-\frac{1}{2\gamma }\le \gamma |g| \le 1+\frac{1}{2\gamma }\\ \gamma g &{}\;\text { if }~\gamma |g| \le 1-\frac{1}{2\gamma }. \end{array}\right. }\nonumber \\ \end{aligned}$$

#### Theorem 3.1

The solution operator $S: \mathbb R^2 \mapsto Y$, which assigns to each pair $(\alpha , \beta ) \in \mathbb R^{2}$ the corresponding solution to the denoising problem (3.1), is Fréchet differentiable and its derivative is characterised by the unique solution $z=S'(\alpha , \beta )[\theta _1, \theta _2] \in Y$ of the following linearised equation:

3.3 $$\begin{aligned}&a(z, \Psi )+ \int _\Omega \theta _1 \ h_\gamma (Du-w)(D \phi - \varphi ) \,\mathrm{d}x\nonumber \\&\quad + \int _\Omega \alpha h'_\gamma (Du-w)(Dz_1-z_2)(D \phi - \varphi ) \,\mathrm{d}x \nonumber \\&\quad + \int _\Omega \theta _2 \ h_\gamma (E w) E \varphi \,\mathrm{d}x\nonumber \\&\quad + \int _\Omega \beta h'_\gamma (E w) E z_2 E \varphi \,\mathrm{d}x \nonumber \\&\quad + \int _\Omega z_1 \phi \,\mathrm{d}x=0, \text { for all } \Psi \in Y. \end{aligned}$$

#### Proof

Thanks to the ellipticity of $a(\cdot , \cdot )$ and the monotonicity of $h_\gamma$, the existence of a unique solution to the linearised equation follows from the Lax-Milgram theorem.

Let $\xi :=y^+- y -z$, where $y=S(\alpha , \beta )$ and $y^+=S(\alpha +\theta _1, \beta + \theta _2)$. Our aim is to prove that $\Vert \xi \Vert _Y= o(|\theta |).$ Combining the equations for $y^+$, *y* and *z* we get that

$$\begin{aligned}&a(\xi , \Psi )+ \int _\Omega (\alpha +\theta _1) \ h_\gamma (Du^+-w^+)(D \phi - \varphi ) \,\mathrm{d}x\\&\quad - \int _\Omega \alpha \ h_\gamma (Du-w)(D \phi - \varphi ) \,\mathrm{d}x\\&\quad - \int _\Omega \theta _1 \ h_\gamma (Du-w)(D \phi - \varphi ) \,\mathrm{d}x\\&\quad - \int _\Omega \alpha h'_\gamma (Du-w)(Dz_1-z_2)(D \phi - \varphi ) \,\mathrm{d}x\\&\quad + \int _\Omega (\beta +\theta _2) h_\gamma (E w^+) E \varphi \,dx- \int _\Omega \beta h_\gamma (E w) E \varphi \,\mathrm{d}x\\&\quad - \int _\Omega \theta _2 \ h_\gamma (E w) E \varphi \,\mathrm{d}x - \int _\Omega \beta \ h'_\gamma (E w) E z_2 E \varphi \,\mathrm{d}x \\&\quad +\,2 \int _\Omega \xi _1 \phi \,dx=0, \text { for all } \Psi \in Y, \end{aligned}$$

where $\xi :=(\xi _1,\xi _2) \in H^1(\Omega ) \times \mathbb H^1(\Omega )$. Adding and subtracting the terms

$$\begin{aligned} \int _\Omega \alpha h'_\gamma (Du-w)(D \delta _u - \delta _w)(D \phi - \varphi ) \,\mathrm{d}x \end{aligned}$$

and

$$\begin{aligned} \int _\Omega \beta h'_\gamma (E w)E \delta _w: E \varphi \,\mathrm{d}x, \end{aligned}$$

where $\delta _u:=u_{\alpha +\theta }-u$ and $\delta _w:=w_{\alpha +\theta }-w$, we obtain that

$$\begin{aligned}&a(\xi , \Psi )+ \int _\Omega \alpha h'_\gamma (Du-w)(D \xi _1- \xi _2)(D \phi - \varphi )\\&\qquad + \int _\Omega \beta h'_\gamma (E w) E \xi _2 :E \varphi \,\mathrm{d}x +2 \int _\Omega \xi _1 \phi \,\mathrm{d}x \\&\quad =- \int _\Omega \alpha \big [ h_\gamma (Du^+-w^+) -h_\gamma (Du-w) \\&\qquad -\, h'_\gamma (Du-w)(D \delta _u - \delta _w)\big ] (D \phi - \varphi )\\&\qquad - \int _\Omega \theta _1 \ \big [ h_\gamma (Du^+-w^+)\\&\qquad -\,h_\gamma (Du-w) \big ] (D \phi - \varphi ) \,\mathrm{d}x \\&\qquad - \,\int _\Omega \beta \left[ h_\gamma (E w^+)-h_\gamma (E w)- h'_\gamma (E w) E \delta _w \right] :E \varphi \,\mathrm{d}x \\&\qquad - \int _\Omega \theta _2 \ \left[ h_\gamma (E w_{\alpha +\theta })-h_\gamma (E w) \right] : E \varphi \,\mathrm{d}x, \text { for all } \Psi \in Y. \end{aligned}$$

Testing with $\Psi =\xi$ and using the monotonicity of $h_\gamma '(\cdot )$, we get that

$$\begin{aligned} \Vert \xi \Vert _Y\le & {} C \left\{ |\alpha | \big \Vert h_\gamma (Du^+-w^+)-h_\gamma (Du-w)\right. \\&-\, h'_\gamma (Du-w)(D \delta _u - \delta _w) \big \Vert _{L^2} \\&+\,|\theta _1| \left\| h_\gamma (Du^+-w^+)-h_\gamma (Du-w) \right\| _{L^2}\\&\left. +\,|\beta | \left\| h_\gamma (E w^+)-h_\gamma (E w)- h'_\gamma (E w) E \delta _w \right\| _{L^2} \right. \\&\left. +\,|\theta _2| \left\| h_\gamma (E w_{\alpha +\theta })-h_\gamma (E w) \right\| _{L^2} \right\} , \end{aligned}$$

for some generic constant $C >0$. Considering the differentiability and Lipschitz continuity of $h_\gamma '(\cdot )$, it then follows that

3.4 $$\begin{aligned} \Vert \xi \Vert _Y\le & {} C \left( |\alpha |~ o (\left\| y^+-y \right\| _{1,p}) +\,|\theta _1| \left\| y_{\alpha +\theta }-y \right\| _{Y} \right. \nonumber \\&+\,\left. |\beta |~ o (\left\| w^+-w \right\| _{1,p}) +\,|\theta _2| \left\| w_{\alpha +\theta }-w \right\| _{\mathbb H^1(\Omega )} \right) ,\nonumber \\ \end{aligned}$$

where $\Vert \cdot \Vert _{1,p}$ stands for the norm in the space $\mathbb W^{1,p}(\Omega )$. From regularity results for second-order systems (see [24, Theorem 1, Remark 14]), it follows that

$$\begin{aligned}&\left\| y^+-y \right\| _{1,p} \\&\quad \le L |\theta | \left( \Vert \mathrm {Div}~ h_\gamma (Du -w)\Vert _{-1,p}+ \Vert h_\gamma (Du -w)\Vert _{-1,p}\right. \\&\qquad \left. +\,\Vert \mathrm {Div}~ h_\gamma (E w)\Vert _{-1,p} \right) \\&\quad \le L |\theta | \left( 2 \Vert h_\gamma (Du -w)\Vert _{L^\infty }+\Vert h_\gamma (E w)\Vert _{L^\infty } \right) \\&\quad \le \widetilde{L} |\theta |, \end{aligned}$$

since $|h_\gamma (\cdot )| \le 1$. Inserting the latter in estimate (3.4), we finally get that

$$\begin{aligned} \Vert \xi \Vert _Y= o(|\theta |). \end{aligned}$$

$$\square $$

#### Remark 3.1

The extra regularity result for second-order systems used in the last proof and due to Gröger [24, Thm. 1, Rem. 14] relies on the properties of the domain $\Omega$. The result was originally proved for $C^2$ domains. However, the regularity of the domain (in the sense of Gröger) may also be verified for convex Lipschitz bounded domains [17], which is precisely our image domain case.

#### Remark 3.2

The Fréchet differentiability proof makes use of the quasilinear structure of the $\text {TGV}^2$ variational form, making it difficult to extend to the ICTV model without further regularisation terms. For the latter, however, a Gâteaux differentiability result may be obtained using the same proof technique as in [22].

### The Adjoint Equation

Next, we use the Lagrangian formalism for deriving the adjoint equations for both the $\text {TGV}^2$ and ICTV learning problems. The existence of a solution to the adjoint equation follows from the Lax-Milgram theorem.

Defining the Lagrangian associated to the $\text {TGV}^2$ learning problem by

$$\begin{aligned}&\mathcal L(u,w,\alpha ,\beta ,p_1,p_2) = F(u) +\mu (u, p_1)_{H^1}+ \mu (w, p_2)_{\mathbb H^1}\\&\quad + \int _\Omega \alpha h_\gamma (Du - w)(D p_1- p_2)\\&\quad + \int _\Omega \beta h_\gamma (E w) E p_2 + \int _\Omega (u-f) p_1, \end{aligned}$$

and taking the derivative with respect to the state variable (*u*, *w*), we get the necessary optimality condition

$$\begin{aligned}&\mathcal L_{(u,w)}'(u,w,\alpha ,\beta ,p_1,p_2)[(\delta _u, \delta _w)]\\&\quad = F'(u)\delta _u +\mu (p_1, \delta _u)_{H^1}+ \mu (p_2, \delta _w)_{\mathbb H^1}\\&\qquad + \int _\Omega \alpha h_\gamma ' (Du - w)(D \delta _u- \delta _w)(D p_1- p_2)\\&\qquad + \int _\Omega \beta h_\gamma ' (E w) E \delta _w E p_2 + \int _\Omega p_1 \delta _u=0. \end{aligned}$$

If $$\delta _w=0$$, then

3.5 $$\begin{aligned}&\mu (p_1, \delta _u)_{H^1}+ \int _\Omega \alpha h_\gamma ' (Du - w)(D p_1- p_2) D \delta _u\nonumber \\&\quad + \int _\Omega p_1 \delta _u=-\nabla _u F(u)\delta _u, ~\text { for all } \delta _u \in H^1(\Omega ), \end{aligned}$$

whereas if $\delta _u=0$, then

3.6 $$\begin{aligned}&\mu (p_2, \delta _w)_{\mathbb H^1} - \int _\Omega \alpha h_\gamma ' (Du - w)(D p_1- p_2) \delta _w\nonumber \\&\quad + \int _\Omega \beta h_\gamma ' (E w) \ E p_2 \ E \delta _w =0, ~\text { for all } \delta _w \in \mathbb H^1(\Omega ).\nonumber \\ \end{aligned}$$

#### Theorem 3.2

Let $(u,w) \in H^1(\Omega ) \times \mathbb H^1(\Omega )$. There exists a unique solution $\Pi =(p_1,p_2) \in Y= H^1(\Omega ) \times \mathbb H^1(\Omega )$ to the adjoint system

3.7 $$\begin{aligned}&\mu (\Pi , \delta _y)_{Y} + \int _\Omega \alpha h_\gamma ' (Du - w)(D \delta _u- \delta _w)(D p_1- p_2)\nonumber \\&\qquad + \int _\Omega \beta h_\gamma ' (E w) E \delta _w E p_2 + \int _\Omega p_1 \delta _v\nonumber \\&\quad =- F'(u)\delta _u, ~\text { for all } \delta _y \in Y. \end{aligned}$$

The corresponding solution is called *adjoint state* associated to (*v*, *w*).

#### Proof

We have to show that the left-hand side of equation (3.7) constitutes a bilinear, continuous and coercive form on $Y \times Y$. Linearity and continuity follows immediately. For the coercivity, let us take $\delta _y = \Pi$. Since $h_\gamma$ is a monotone function, the terms $\int _\Omega \alpha h_\gamma ' (Du - w)(D p_1- p_2)(D p_1- p_2)$ and $\int _\Omega \beta h_\gamma ' (E w) E p_2 E p_2$ become positive, yielding

$$\begin{aligned}&\mu \Vert \Pi \Vert ^2_{Y} + \int _\Omega \alpha h_\gamma ' (Du - w)(D p_1- p_2)(D p_1- p_2)\\&\quad + \int _\Omega \beta h_\gamma ' (E w) E p_2 E p_2 + \int _\Omega p_1^2 \ge \mu \Vert \Pi \Vert ^2_{Y}. \end{aligned}$$

Thus, coercivity holds and, using Lax-Milgram theorem, we conclude that there exists a unique solution to the adjoint system (3.7). $\square$

#### Remark 3.3

For the ICTV model, it is possible to proceed formally with the Lagrangian approach. We recall that a necessary and sufficient optimality condition for the ICTV functional is given by

3.8 $$\begin{aligned}&\mu (u, \phi )_{H^1}{+} \mu (\nabla v, \nabla \varphi )_{\mathbb H^1} + \int _\Omega \alpha h_\gamma (Du {-} \nabla v)(D \phi {-} \nabla \varphi )\nonumber \\&\quad + \int _\Omega \beta h_\gamma (D \nabla v) D \nabla \varphi + \int _\Omega (u-f)\phi =0, \nonumber \\&\quad \text { for all }(\phi , \varphi ) \in H^1(\Omega ) \times \mathbb H^1(\Omega ) \end{aligned}$$

and the correspondent Lagrangian functional $\mathcal L$ is given by

$$\begin{aligned}&\mathcal L(u,v,\alpha ,\beta ,p_1,p_2) = F(u) +\mu (u, p_1)_{H^1}\\&\quad + \mu (\nabla v, \nabla p_2)_{\mathbb H^1}+ \int _\Omega \alpha h_\gamma (Du - \nabla v)(D p_1- \nabla p_2) \\&\quad + \int _\Omega \beta h_\gamma (D \nabla v) D \nabla p_2+ \int _\Omega (u-f) p_1. \end{aligned}$$

Deriving the Lagrangian with respect to the state variables (*u*, *v*) and setting it equal to zero yields

$$\begin{aligned}&\mathcal L_{(u,v)}'(u,v,\alpha ,\beta ,p_1,p_2)[(\delta _u, \delta _v)]\\&\quad = F'(u)\delta _u +\mu (p_1, \delta _u)_{H^1}+ \mu (\nabla p_2, \nabla \delta _v)_{\mathbb H^1}\\&\qquad + \int _\Omega \alpha h_\gamma ' (Du - \nabla v)(D \delta _u- \nabla \delta _v)(D p_1- \nabla p_2)\\&\qquad + \int _\Omega \beta h_\gamma ' (D \nabla v) D \nabla \delta _v D \nabla p_2 + \int _\Omega p_1 \delta _u=0. \end{aligned}$$

By taking successively $\delta _v=0$ and $\delta _u=0$, the following adjoint system is obtained

3.9a $$\begin{aligned}&\mu (p_1, \delta _u)_{H^1}+ \int _\Omega \alpha h_\gamma ' (Du - \nabla v)(D p_1- \nabla p_2) D \delta _u\nonumber \\&\quad + \int _\Omega p_1 \delta _u=-F'(u)\delta _u, \end{aligned}$$

3.9b $$\begin{aligned}&\mu (\nabla p_2, \nabla \delta _v)_{\mathbb H^1} + \int _\Omega \alpha h_\gamma ' (Du - \nabla v)(D p_1- \nabla p_2) \nabla \delta _v\nonumber \\&\quad + \int _\Omega \beta h_\gamma ' (D \nabla v) D \nabla p_2 D \nabla \delta _v =0. \end{aligned}$$

### Optimality Condition

Using the differentiability of the solution operator and the well-posedness of the adjoint equation, we derive next an optimality system for the characterisation of local minima of the bilevel learning problem. Besides the optimality condition itself, a gradient formula arises as byproduct, which is of importance in the design of solution algorithms for the learning problems.

#### Theorem 3.3

Let $(\bar{\alpha }, \bar{\beta }) \in \mathbb R^2_+$ be a local optimal solution for problem (2.3). Then there exist Lagrange multipliers $\Pi \in Y:=H^1(\Omega ) \times \mathbb H^1(\Omega )$ and $\lambda _1, \lambda _2 \in \mathbb R$ such that the following system holds

3.10a $$\begin{aligned}&a(y, \Psi )+\alpha \int _\Omega h_\gamma (Du-w)(D \phi - \varphi ) \,\mathrm{d}x\nonumber \\&\quad +\, \beta \int _\Omega h_\gamma (E w) E \varphi \,\mathrm{d}x + \int _\Omega (u-f)\phi \,\mathrm{d}x=0, \text { for all }\nonumber \\&\quad \Psi =(\phi , \varphi ) \in Y, \end{aligned}$$

3.10b $$\begin{aligned}&a(\Pi , \Psi )+\alpha \int _\Omega h_\gamma ' (Du-w)(D p_1-p_2)(D \phi - \varphi ) \,\mathrm{d}x\nonumber \\&\quad +\, \beta \int _\Omega h_\gamma ' (E w) \ E p_2 \ E \varphi \,\mathrm{d}x + \int _\Omega p_1 \phi \,dx=-F_u(u)[\phi ],\nonumber \\&\quad \text { for all } \Psi =(\phi , \varphi ) \in Y, \end{aligned}$$

3.10c $$\begin{aligned}&\lambda _1= \int _\Omega h_\gamma (Du-w)(D p_1 -p_2), \end{aligned}$$

3.10d $$\begin{aligned}&\lambda _2= \int _\Omega h_\gamma (Ew), \ E p_2 \end{aligned}$$

3.10e $$\begin{aligned}&\lambda _1 \ge 0, \qquad \lambda _2 \ge 0, \end{aligned}$$

3.10f $$\begin{aligned}&\lambda _1 \cdot \bar{\alpha }= \lambda _2 \cdot \bar{\beta }=0. \end{aligned}$$

#### Proof

Consider the reduced cost functional $\mathcal F(\alpha , \beta )=F(u(\alpha , \beta )).$ The bilevel optimisation problem can then be formulated as

$$\begin{aligned}&\min _{(\alpha , \beta ) \in C} \mathcal F(\alpha , \beta ), \end{aligned}$$

where $\mathcal F: \mathbb R^{2} \rightarrow \mathbb R$ and *C* corresponds to the positive orthant in $\mathbb R^2$. From [47, Thm. 3.1], there exist multipliers $\lambda _1, \lambda _2 \in \mathbb R$ such that

$$\begin{aligned}&\lambda _1= \nabla _\alpha \mathcal F(\bar{\alpha }, \bar{\beta }),\\&\lambda _2= \nabla _\beta \mathcal F(\bar{\alpha }, \bar{\beta }),\\&\lambda _1 \ge 0, \quad \lambda _2 \ge 0,\\&\lambda _1 \cdot \bar{\alpha }= \lambda _2 \cdot \bar{\beta }=0. \end{aligned}$$

By taking the derivative with respect to $(\alpha , \beta )$ and denoting by *z* the solution to the linearised equation (3.3), we get, together with the adjoint equation (3.10b), that

$$\begin{aligned} \mathcal F'(\alpha , \beta )[\theta _1,\theta _2]&=F_u(u)z_1= -a(\Pi ,z)\\&\quad \ - \alpha \int _\Omega h_\gamma '(Du-w)(D p_1 {-}p_2 )(D z_1{-}z_2)\\&\quad \ -\beta \int _\Omega h_\gamma '(E w) E p_2 \ E z_2 - \int _\Omega p_1 z_1\\&= -a(z,\Pi ) \\&\quad \ - \alpha \int _\Omega h_\gamma '(Du-w)(D z_1{-}z_2) (D p_1 {-}p_2 )\\&\quad \ -\beta \int _\Omega h_\gamma '(E w) E z_2 \ E p_2- \int _\Omega z_1 p_1 \end{aligned}$$

which, taking into account the linearised equation, yields

3.11 $$\begin{aligned}&\mathcal F'(\alpha , \beta )[\theta _1,\theta _2]=\theta _1 \int _\Omega h_\gamma (Du-w)(D p_1 -p_2 )\nonumber \\&\quad + \,\theta _2 \int _\Omega h_\gamma (E w)E p_2. \end{aligned}$$

Altogether we proved the result.$\square$

#### Remark 3.4

From the existence result (see Remark 2.2), we actually know that, under some assumptions on *F*, $\bar{\alpha }$ and $\bar{\beta }$ are strictly greater than zero. This implies that the multipliers $\lambda _1$ and $\lambda _2$ may be zero, and the problem becomes an unconstrained one. This plays an important role in the design of solution algorithms, since only a mild treatment of the constraints has to be taken into account, as shown in Sect. 6.

## Numerical Algorithms

In this section, we propose a second-order quasi-Newton method for the solution of the learning problem with scalar regularisation parameters. The algorithm is based on a BFGS update, preserving the positivity of the iterates through the line search strategy and updating the matrix cyclically depending on the satisfaction of the curvature condition. For the solution of the lower level problem, a semismooth Newton method with a properly modified Jacobi matrix is considered. Moreover, warm initialisation strategies have to be taken into account in order to get convergence for the $\text {TGV}^2$ problem.

### BFGS Algorithm

Thanks to the gradient characterisation obtained in Theorem 3.3, we next devise a BFGS algorithm to solve the bilevel learning problems with higher-order regularisers. We employ a few technical tricks to ensure convergence of the classical method. In particular, we limit the step length to get at most a fraction closer to the boundary. As shown in [19], the solution is in the interior for the regularisation and cost functionals we are interested in.

Moreover, the good behaviour of the BFGS method depends upon the BFGS matrix staying positive definite. This would be ensured by the Wolfe conditions, but because of our step length limitation, the curvature condition is not necessarily satisfied. (The Wolfe conditions are guaranteed to be satisfied for some step length $\sigma$, if our domain is unbounded, but the range, where the step satisfies the criterion, may be beyond our maximum step length and is not necessarily satisfied closer to the current point.) Instead, we skip the BFGS update if the curvature is negative.

Overall, our learning algorithm may be written as follows:

#### Algorithm 4.1

(BFGS for denoising parameter learning) Pick Armijo line search constant *c*, and target residual $\rho$. Pick initial iterate $(\alpha ^0,\beta ^0)$. Solve the denoising problem (2.3b) for $(\alpha ,\beta )=(\alpha ^0,\beta ^0)$, yielding $u^0$. Initialise $B^1=I$. Set $i :=0$, and iterate the following steps:

1. Solve the adjoint equation (3.10b) for $\Pi ^i$, and calculate $\nabla \mathcal F (\alpha ^i,\beta ^i)$ from (3.11).
2. If $i \ge 2$, do the following:
  - Set $$s :=(\alpha ^i,\beta ^i)-(\alpha ^{i-1}, \beta ^{i-1})$$, and $$r :=\nabla \mathcal F(\alpha ^i,\beta ^i)-\nabla \mathcal F(\alpha ^{i-1},\beta ^{i-1})$$.
  - Perform the BFGS update
    $$\begin{aligned} B^i :={\left\{ \begin{array}{ll} B^{i-1}, &{} s^T r \le 0,\\ B^{i-1} - \frac{(B^{i-1} s) (B^{i-1}s)^T}{t^T B^{i-1} s} + \frac{r r^T}{s^Tr} &{} s^T r > 0. \end{array}\right. } \end{aligned}$$
3. Compute $$\delta _{\alpha , \beta }$$ from
  $$\begin{aligned} B^i \delta _{\alpha , \beta } = g^i. \end{aligned}$$
4. Initialise $$\sigma :=\min \{1, \sigma _{\max }/2\}$$, where
  $$\begin{aligned} \sigma _{\max } :=\max \{ \sigma \ge 0 \mid (\alpha ^i, \beta ^i)+\sigma \delta _{\alpha , \beta } > 0\}. \end{aligned}$$
  Repeat the following:
  - Let $(\alpha _\sigma , \beta _\sigma ) :=(\alpha ^i, \beta ^i)+\sigma \delta _{\alpha , \beta }$, and solve the denoising problem (2.3b) for $(\alpha , \beta )=(\alpha _\sigma , \beta _\sigma )$, yielding $u_\sigma$.
  - If the residual $\Vert (\alpha _\sigma , \beta _\sigma ) - (\alpha ^i, \beta ^i)\Vert /\Vert (\alpha _\sigma , \beta _\sigma )\Vert < \rho$, do the following:
    (i) If $\min _\sigma \mathcal F(\alpha _\sigma , \beta _\sigma ) < \mathcal F(\alpha ^i, \beta ^i)$ over all $\sigma$ tried, choose $\sigma ^*$ the minimiser, set $(\alpha ^{i+1}, \beta ^{i+1}) :=(\alpha _{\sigma ^*}, \beta _{\sigma ^*})$, $u^{i+1} :=u_{\sigma ^*}$, and continue from Step 5.
    (ii) Otherwise end the algorithm with solution $(\alpha ^*, \beta ^*) :=(\alpha ^i, \beta ^i)$.
  - Otherwise, if Armijo condition $\mathcal F(\alpha _\sigma , \beta _\sigma ) \le \mathcal F(\alpha ^i, \beta ^i) + \sigma c \nabla \mathcal F(\alpha ^i,\beta ^i)^T \delta _{\alpha , \beta }$ holds, set $(\alpha ^{i+1}, \beta ^{i+1}) :=(\alpha _{\sigma }, \beta _{\sigma })$, $u^{i+1} :=u_{\sigma }$, and continue from Step 5.
  - In all other cases, set $\sigma :=\sigma /2$ and continue from Step 4a.
5. If the residual $\Vert (\alpha ^{i+1}, \beta ^{i+1}) - (\alpha ^i, \beta ^i)\Vert /\Vert (\alpha ^{i+1}, \beta ^{i+1})\Vert < \rho$, end the algorithm with $(\alpha ^* , \beta ^*) :=(\alpha ^{i+1}, \beta ^{i+1})$. Otherwise continue from Step 1 with $i :=i+1$.

Step (4) ensures that the iterates remain feasible, without making use of a projection step.

### An Infeasible Semismooth Newton Method

In this section, we consider semismooth Newton methods for solving the $\text {TGV}^2$ and the ICTV denoising problems. Semismooth Newton methods feature a local superlinear convergence rate and have been previously successfully applied to image processing problems (see, e.g. [21, 29, 32]). The primal-dual algorithm we use here is an extension of the method proposed in [29] to the case of higher-order regularisers.

In variational form, the $\text {TGV}^2$ denoising problem can be written as

$$\begin{aligned}&\mu \int _\Omega (Du \cdot D \phi + v \phi )+ \int _\Omega \alpha h_\gamma (Du - w) D \phi \\&\quad + \int _\Omega (u-f) \phi =0, \quad \forall \phi \in H^1(\Omega )\\&\mu \int _\Omega (Ew : E \varphi + w \varphi ) - \int _\Omega \alpha h_\gamma (Du - w) D \varphi \\&\quad + \int _\Omega \beta h_\gamma (E w) \ E \varphi =0, \quad \forall \varphi \in \mathbb H^1(\Omega ) \end{aligned}$$

or, in general abstract primal-dual form, as

4.1a $$\begin{aligned}&L y + \sum _{i=1}^{N} A_j^* q_j = f \quad \text { in } \Omega , \end{aligned}$$

4.1b $$\begin{aligned}&\max \{1/\gamma , |[A_j y](x)|_2\} q_j(x) - \alpha _j [A_j y](x) = 0 \text { a.e. in }\Omega , \nonumber \\&\quad j=1,\ldots ,N. \end{aligned}$$

where $L \in \mathcal L (H^{1}(\Omega ; \mathbb {R}^m),H^{1}(\Omega ; \mathbb {R}^m)')$ is a second-order linear elliptic operator, $A_j, ~j=1, \dots , N$, are linear operators acting on *y* and $q_j(x), ~j=1, \dots , N$, correspond to the dual multipliers.

Let us set

$$\begin{aligned} \mathfrak {m}_j(y) :=\max \{1/\gamma , |[A_j y](x)|_2\}. \end{aligned}$$

Let us also define the diagonal application $\mathfrak {D}(y): L^2(\Omega ; \mathbb {R}^{m}) \rightarrow L^2(\Omega ; \mathbb {R}^{m})$ by

$$\begin{aligned}{}[\mathfrak {D}(y) q](x) = y(x) q(x), \quad (x \in \Omega ) \end{aligned}$$

We may derive $\nabla _y [\mathfrak {D}(\mathfrak {m}_j(y)) q_j]$ being defined by

$$\begin{aligned}&\nabla _y [\mathfrak {D}(\mathfrak {m}_j(y)) p_j] = A_j^* \mathfrak {D}(q_j) \mathfrak {N}(A_j y)\\&\quad \text {where} \quad \mathfrak {N}(z) :={\left\{ \begin{array}{ll} 0, &{} |z(x)|_2 < 1/\gamma \\ \frac{z(x)}{|z(x)|_2}, &{} |z(x)|_2 \ge 1/\gamma . \\ \end{array}\right. } \end{aligned}$$

Then (4.1a), (4.1b) may be written as

$$\begin{aligned}&L y + \sum _{i=1}^{N} A_j^* q_j = f \quad \text { in } \Omega \\&\quad \mathfrak {D}(\mathfrak {m}_j(y)) q_j{-} \alpha _j A_j y {=} 0, \quad \text {a.e. in } \Omega , \quad (j=1,\ldots ,N). \end{aligned}$$

Linearising, we obtain the system

SSN-1 $$\begin{aligned} \begin{pmatrix} L &{} A_1^* &{} \ldots &{} A_N^* \\ - \alpha _1 A_1 + \mathfrak {N}(A_1 y) \mathfrak {D}(q_1) A_1 &{} \mathfrak {D}(\mathfrak {m}_j(y)) &{} 0 &{} 0 \\ \vdots &{} 0 &{} \ddots &{} 0 \\ - \alpha _NA_N+ \mathfrak {N}(A_Ny) \mathfrak {D}(q_N) A_N&{} 0 &{} 0 &{} \mathfrak {D}(\mathfrak {m}_N(y)) \\ \end{pmatrix} \begin{pmatrix} \delta y \\ \delta q_1 \\ \vdots \\ \delta q_N\\ \end{pmatrix} = R \end{aligned}$$

where

$$\begin{aligned} R :=\begin{pmatrix} -L y - \sum _{i=1}^{N} A_j^* q_j + f \\ \alpha _1 A_1 y - \mathfrak {D}(\mathfrak {m}_1(y)) q_1\\ \vdots \\ \alpha _NA_Ny - \mathfrak {D}(\mathfrak {m}_N(y)) q_N\end{pmatrix}. \end{aligned}$$

The semismooth Newton method solves (SSN-1) at a current iterate $(y^i, q_1^i, \ldots q_N^i)$. It then updates

SSN-2 $$\begin{aligned}&(y^{i+1}, \widetilde{q}_1^{i+1}, \ldots \widetilde{q}^{i+1}_N)\nonumber \\&\quad := (y^i + \tau \delta y, q_1^i + \tau \delta q_1, q_N^i + \tau \delta q_N), \end{aligned}$$

for a suitable step length $\tau$, allowing $\widetilde{q}^{i+1}$ to become infeasible in the process. That is, it may hold that $|\widetilde{q}_j^{i+1}(x)|_2 > \alpha _j$, which may lead to non-descent directions. In order to globalise the method, one projects

SSN-3 $$\begin{aligned}&q_j^{i+1} :=\mathfrak {P}(\widetilde{q}_j^{i+1}; \alpha _j), \quad \text {where} \quad \mathfrak {P}(q, \alpha )(x)\\&\quad :={{\mathrm{sgn}}}(q(x)) \min \{\alpha , |q(x)|\}, \end{aligned}$$

in the building of the Jacobi matrix. Following [29, 42], it can be shown that a discrete version of the method (SSN-1)–(SSN-3) converges globally and locally superlinearly near a point where the subdifferentials of the operator on $(y, q_1, \ldots q_N)$ corresponding (4.1) are non-singular. Further dampening as in [29] guarantees local superlinear convergence at any point. We do not present the proof, as going into the discretisation and dampening details would expand this work considerably.

#### Remark 4.1

The system (SSN-1) can be further simplified, which is crucial to obtain acceptable performance with $\text {TGV}^2$. Indeed, observe that *B* is invertible, so we may solve $\delta u$ from

4.2 $$\begin{aligned} B \delta y = R_1 - \sum _{j=1}^N A_j^* \delta q_j. \end{aligned}$$

Thus, we may simplify $\delta y$ out of (SSN-1) and only solve for $\delta q_1, \ldots , \delta q_N$ using a reduced system matrix. Finally, we calculate $\delta y$ from (4.2).

For the denoising sub-problem (2.3b), we use the method (SSN-1)–(SSN-3) with the reduced system matrix of Remark 4.1. Here, we denote by *y* in the case of TGV$^2$ the parameters

$$\begin{aligned} y=(u,w), \end{aligned}$$

and in the case of ICTV

$$\begin{aligned} y=(u,v). \end{aligned}$$

For the calculation of the step length $\tau$, we use Armijo line search with parameter $c=1{\textsc {e}}^{-4}$. We end the SSN iterations when

$$\begin{aligned} \tau \frac{\Vert \delta Y^i\Vert }{\max \{1,\Vert Y^i\Vert \}} \le 1{\textsc {e}}^{-5}, \end{aligned}$$

where $$\delta Y^i:=(\delta y^i,\delta q_1^i, \ldots , \delta q_N^i)$$, and $$Y^i:=(y^i, q_1^i, \ldots , q_N^i)$$.

**Table 1:** Quantified results for the parrot image ($\ell =256=\text {image width/height in pixels}$)

Denoise	Cost	Initial ($\alpha$,$\beta$)	Result ($\alpha$*, $\beta$*)	Cost	SSIM	PSNR	Its.	Fig.
$\hbox {TGV}^{2}$	$L_\eta ^1 \nabla$	$(\alpha _{\mathrm{TV}}^*/\ell ,\alpha _{\mathrm{TV}}^*)$	(0.069/$\ell ^{2}$, 0.051/ $\ell$)	6.615	0.897	31.720	12	4c
$$\hbox {TGV}^{2}$$	$$L_2^2 $$	$$(\alpha _{\mathrm{TV}}^*/\ell ,\alpha _{\mathrm{TV}}^*)$$	(0.058/$$\ell ^{2}$$, 0.041/$$\ell $$)	6.412	0.890	31.992	11	4d
ICTV	$L_\eta ^1 \nabla$	$(\alpha _{\mathrm{TV}}^*/\ell ,\alpha _{\mathrm{TV}}^*)$	(0.068/ $\ell ^{2}$, 0.051/$\ell$)	6.656	0.895	31.667	16	4e
ICTV	$$L_2^2 $$	$$(\alpha _{\mathrm{TV}}^*/\ell ,\alpha _{\mathrm{TV}}^*)$$	(0.051/$$\ell ^{2}$$, 0.041/$$\ell $$)	6.439	0.887	31.954	7	4f
TV	$L_\eta ^1 \nabla$	$0.1/\ell$	0.057/$\ell$	6.944	0.887	31.298	10	4g
TV	$$L_2^2 $$	$$0.1/\ell $$	0.042/$$\ell $$	6.623	0.879	31.710	12	4h

### Warm Initialisation

In our numerical experimentation, we generally found Algorithm 4.1 to perform well for learning the regularisation parameter for $\text {TV}$ denoising as was done in [22]. For learning the two (or even more) regularisation parameters for $\text {TGV}^2$ denoising, we found that a warm initialisation is needed to obtain convergence. More specifically, we use $\text {TV}$ as an aid for discovering both the initial iterate $(\alpha ^0,\beta ^0)$ as well as the initial BFGS matrix $B^1$. This is outlined in the following algorithm:

#### Algorithm 4.2

(BFGS initialisation for $\text {TGV}^2$ parameter learning) Pick a heuristic factor $\delta _0 > 0$. Then do the following:

1. Solve the corresponding problem for $\text {TV}$ using Algorithm 4.1. This yields optimal $\text {TV}$ denoising parameter $\alpha _\text {TV}^*$, as well as the BFGS estimate $B_\text {TV}$ for $\nabla ^2 \mathcal F (\alpha _\text {TV}^*)$.
2. Run Algorithm 4.1 for $\text {TGV}^2$ with initialisation $(\alpha ^0,\beta ^0) :=(\alpha _\text {TV}^* \delta _0, \alpha _\text {TV}^*)$, and initial BFGS matrix $B^1 :=\mathrm {diag}(B_\text {TV}\delta _0, B_\text {TV})$.

With $\Omega =(0, 1)^2$, we pick $\delta _0=1/\ell$, where the original discrete image has $\ell \times \ell$ pixels. This corresponds to the heuristic [2, 44] that if $\ell \approx 128$ or 256, and the discrete image is mapped into the corresponding domain $\Omega =(0, \ell )^2$ directly (corresponding to spatial step size of one in the discrete gradient operator), then $\beta \in (\alpha , 1.5 \alpha )$ tends to be a good choice. We will later verify this through the use of our algorithms. Now, if $f \in \text {BV}((0, \ell )^2)$ is rescaled to $\text {BV}((0, 1)^2)$, i.e. $\widetilde{f}(x) :=f(x/\ell )$, then with $\widetilde{u}(x) :=u(x/\ell )$ and $\widetilde{w}(x) :=w(x/\ell )/\ell$, we have the theoretical equivalence

4.3 $$\begin{aligned}&\frac{1}{2}\Vert f-u\Vert _{L^2((0, \ell )^2)}^2 +\alpha \Vert Du-w\Vert _{\mathcal {M}((0, \ell )^2; \mathbb {R}^2)}\nonumber \\&\qquad +\beta \Vert Ew\Vert _{\mathcal {M}((0, \ell )^2; \mathbb {R}^{2 \times 2})} \end{aligned}$$

4.4 $$\begin{aligned}&\quad = n^2\left( \frac{1}{2}\Vert \widetilde{f}-\widetilde{u}\Vert _{L^2((0,1)^2)}^2 +n\alpha \Vert D\widetilde{u}-\widetilde{w}\Vert _{\mathcal {M}((0,1)^2; \mathbb {R}^2)}\right. \nonumber \\&\qquad \left. +n^2\beta \Vert E\widetilde{w}\Vert _{\mathcal {M}((0,1)^2; \mathbb {R}^{2 \times 2})} \right) . \end{aligned}$$

This introduces the factor $1/\ell =|\Omega |^{-1/2}$ between rescaled $\alpha$, $\beta$.

**Table 2:** Quantified results for the synthetic image ($\ell =256=\text {image width/height in pixels}$)

Denoise	Cost	Initial $\vec \alpha$	Result $\vec \alpha ^*$	Value	SSIM	PSNR	Its.	Fig.
TGV$^{2}$	$L_\eta ^1 \nabla$	$(\alpha _{\mathrm{TV}}^*/\ell ,\alpha _{\mathrm{TV}}^*)$	(0.453/$\ell ^{2}$, 0.071/$\ell$)	3.769	0.989	36.606	17	5c
TGV$$^{2}$$	$$L_2^2 $$	$$(\alpha _{\mathrm{TV}}^*/\ell ,\alpha _{\mathrm{TV}}^*)$$	(0.307/$$\ell ^{2}$$, 0.055/$$\ell $$)	3.603	0.986	36.997	19	5d
ICTV	$L_\eta ^1 \nabla$	$(\alpha _{\mathrm{TV}}^*/\ell ,\alpha _{\mathrm{TV}}^*)$	(0.505/$\ell ^{2}$, 0.103/$\ell$)	4.971	0.970	34.201	23	5e
ICTV	$$L_2^2 $$	$$(\alpha _{\mathrm{TV}}^*/\ell ,\alpha _{\mathrm{TV}}^*)$$	(0.056/$$\ell ^{2}$$, 0.049/$$\ell $$)	3.947	0.965	36.206	7	5f
TV	$L_\eta ^1 \nabla$	$0.1/\ell$	0.136/$\ell$	5.521	0.966	33.291	6	5g
TV	$$L_2^2 $$	$$0.1/\ell $$	0.052/$$\ell $$	4.157	0.948	35.756	7	5h

**Fig. 3:**
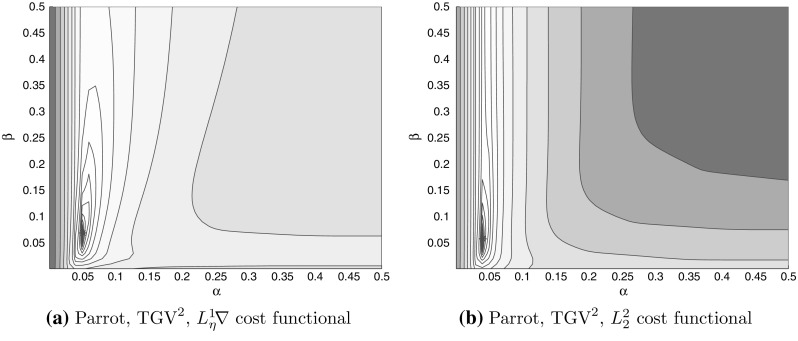
Cost functional value versus $(\alpha , \beta )$ for $\text {TGV}^2$ denoising, for the parrot test images, for both $L_2^2$ and $L_\eta ^1\!\nabla$ cost functionals. The illustrations are contour plots of function value versus $(\alpha , \beta )$

**Fig. 4:**
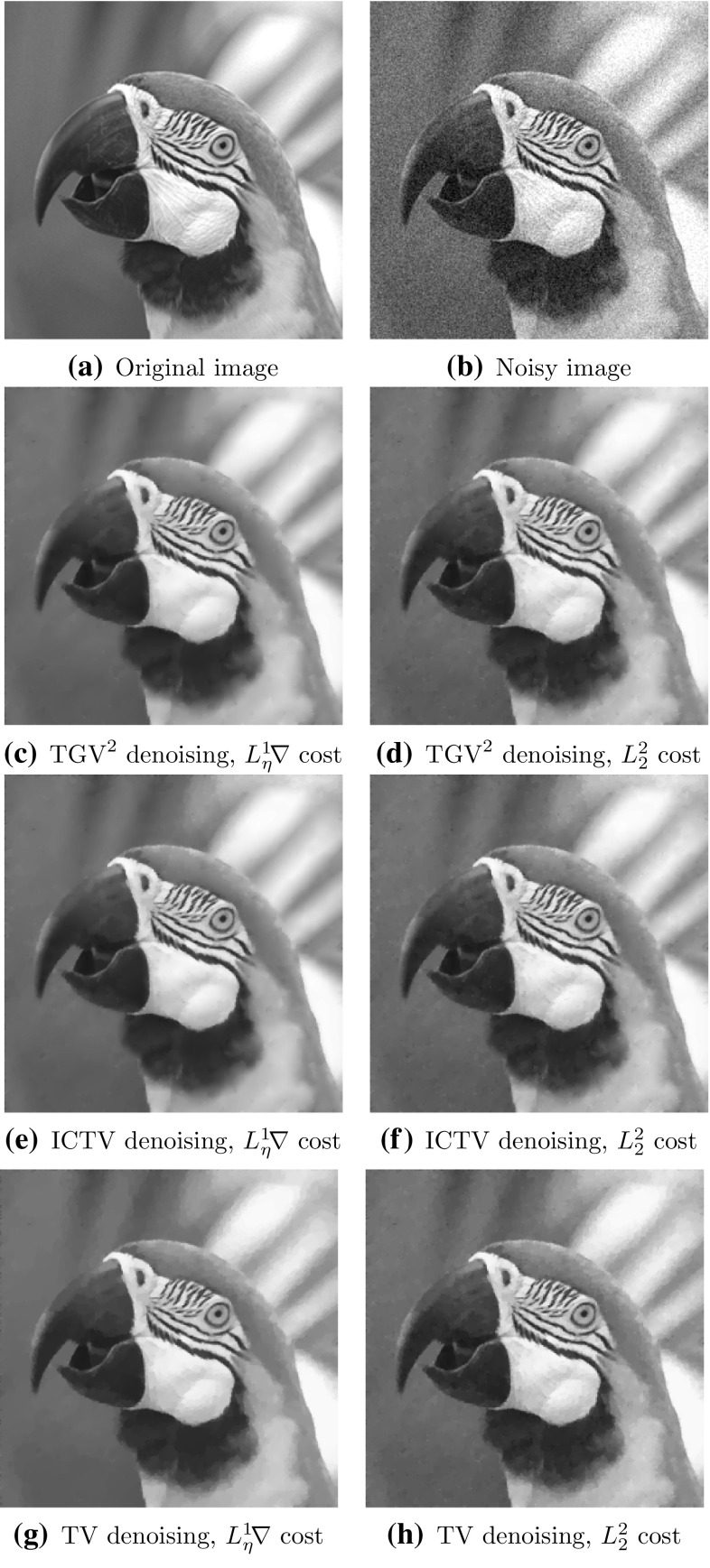
Optimal denoising results for initial guess $\vec \alpha =(\alpha _{\text {TV}}^*/\ell , \alpha _{\text {TV}}^*)$ for $\text {TGV}^2$ and $\vec \alpha =0.1/\ell$ for $\text {TV}$

**Fig. 5:**
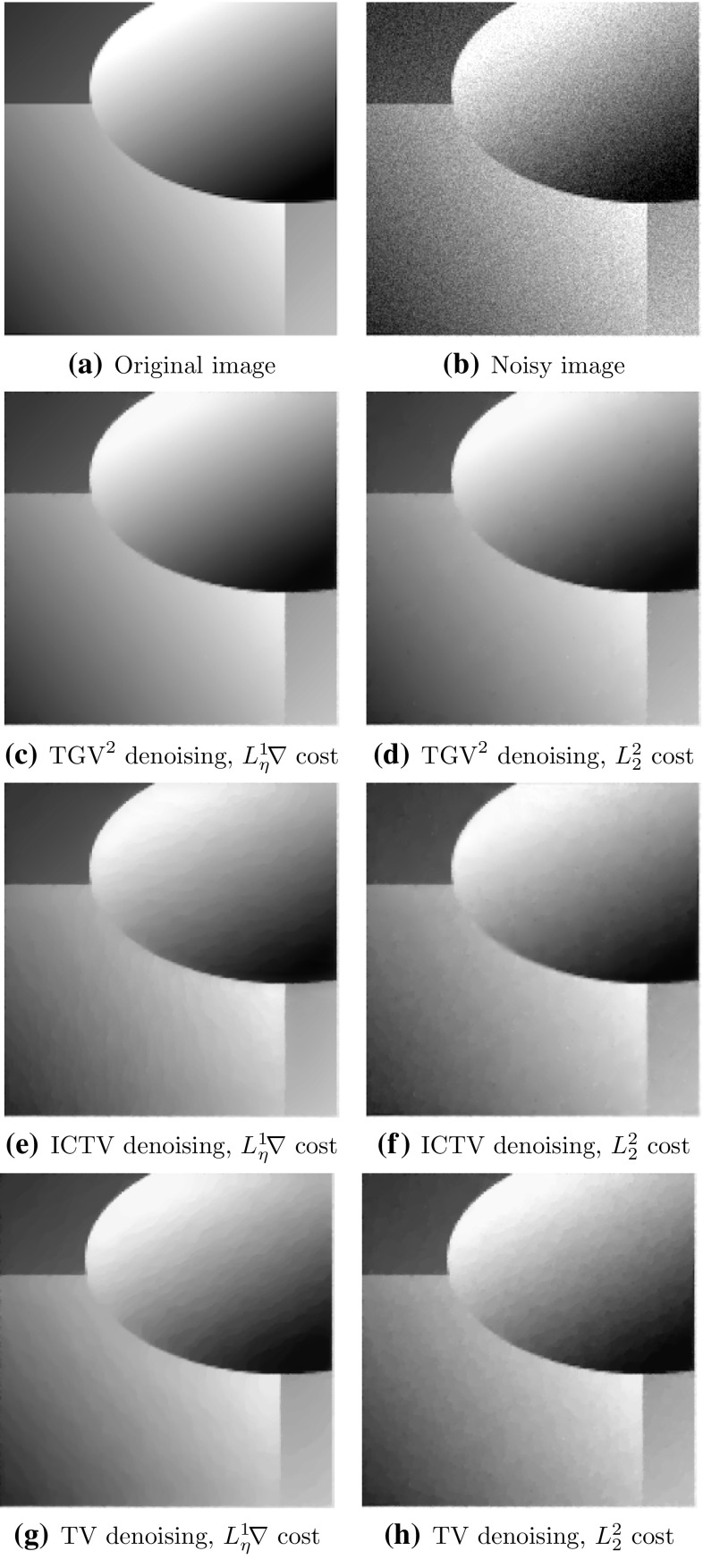
Optimal denoising results for initial guess $\vec \alpha =(\alpha _{\text {TV}}^*/\ell , \alpha _{\text {TV}}^*)$ for $\text {TGV}^2$ and $\vec \alpha =0.2/\ell$ for $\text {TV}$

## Experiments

In this section, we present some numerical experiments to verify the theoretical properties of the bilevel learning problems and the efficiency of the proposed solution algorithms. In particular, we exhaustively compare the performance of the new proposed cost functional with respect to well-known quality measures, showing a better behaviour of the new cost for the chosen tested images. The performance of the proposed BFGS algorithm, combined with the semismooth Newton method for the lower level problem, is also examined.

Moreover, on basis of the learning setting proposed, a thorough comparison between $\text {TGV}^2$ and $\text {ICTV}$ is carried out. The use of higher-order regularisers in image denoising is rather recent, and the question on whether $\text {TGV}^2$ or ICTV performs better has been around. We target that question and, on basis of the bilevel learning approach, we are able to give some partial answers.

### Gaussian Denoising

We tested Algorithm 4.1 for $\text {TV}$ and Algorithm 4.2 for $\text {TGV}^2$ Gaussian denoising parameter learning on various images. Here we report the results for two images, the parrot image in Fig. 4a, and the geometric image in Fig. 5. We applied synthetic noise to the original images, such that the PSNR of the parrot image are 24.7, and the PSNR of the geometric image is 24.8.

In order to learn the regularisation parameter $\alpha$ for $\text {TV}$, we picked initial $\alpha ^0=0.1/\ell$. For $\text {TGV}^2$, initialisation by $\text {TV}$ was used as in Algorithm 4.1. We chose the other parameters of Algorithm 4.1 as $c=1{\textsc {e}}^{-4}$, $\rho =1{\textsc {e}}^{-5}$, $\theta =1{\textsc {e}}{-8}$, and $\Theta =10$. For the SSN denoising method, the parameters $\gamma =100$ and $\mu =1{\textsc {e}}^{-10}$ were chosen.

We have included results for both the $L^2$-squared cost functional ${L_2^2}$ and the Huberised total variation cost functional ${L_\eta ^1\!\nabla }$. The learning results are reported in Table 1 for the parrot images, and Table 2 for the geometric image. The denoising results with the discovered parameters are shown in Figs 4 and 5. We report the resulting optimal parameter values, the cost functional value, PSNR, SSIM [46], as well as the number of iterations taken by the outer BFGS method.

Our first observation is that all approaches successfully learn a denoising parameter that gives a good-quality denoised image. Secondly, we observe that the gradient cost functional ${L_\eta ^1\!\nabla }$ performs visually and in terms of SSIM significantly better for $\text {TGV}^2$ parameter learning than the cost functional ${L_2^2}$. In terms of PSNR, the roles are reversed, as should be, since the ${L_2^2}$ is equivalent to PSNR. This again confirms that PSNR is a poor-quality measure for images. For $\text {TV}$, there is no significant difference between different cost functionals in terms of visual quality, although the PSNR and SSIM differ.

We also observe that the optimal $\text {TGV}^2$ parameters $(\alpha ^*, \beta ^*)$ generally satisfy $\beta ^*/\alpha ^* \in (0.75, 1.5)/\ell$. This confirms the earlier observed heuristic that if $\ell \approx 128,\, 256$ then $\beta \in (1, 1.5) \alpha$ tends to be a good choice. As we can observe from Figs. 4 and 5, this optimal $\text {TGV}^2$ parameter choice also avoids the staircasing effect that can be observed with $\text {TV}$ in the results.

In Fig. 3, we have plotted by the red star the discovered regularisation parameter $(\alpha ^*, \beta ^*)$ reported in Fig. 4. Studying the location of the red star, we may conclude that Algorithms 4.1 and 4.2 manage to find a nearly optimal parameter in very few BFGS iterations.

### Statistical Testing

**Fig. 6:**
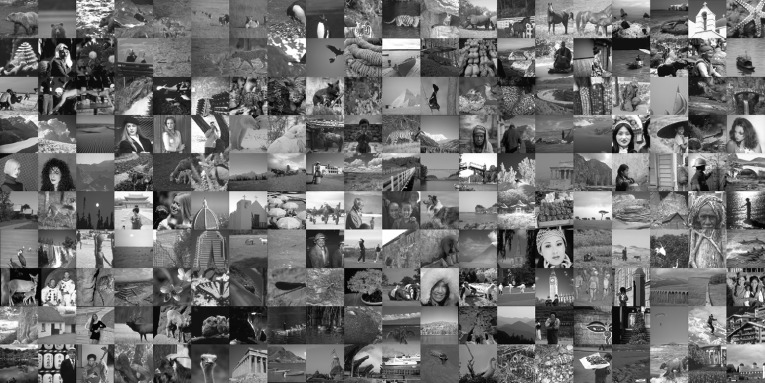
The 200 images of the Berkeley segmentation dataset BSDS300 [36], cropped to be rectangular, keeping *top left corner*, and resized to $128 \times 128$

**Fig. 7:**
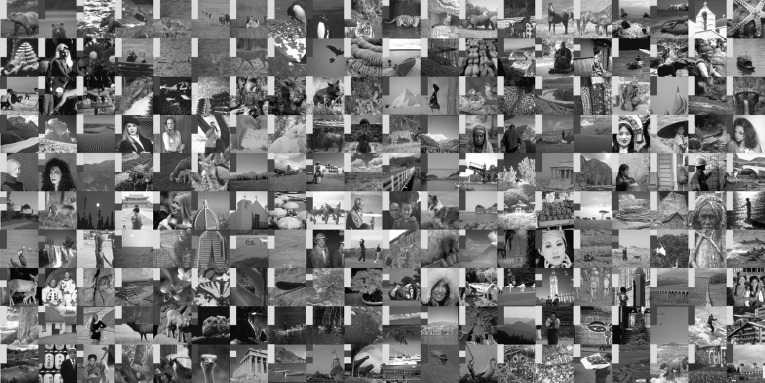
Ordering of regularisers with individual learning, $L_\eta ^1 \nabla$ cost, and noise variance $\sigma ^{2}=2$, on the 200 images of the BSDS300 dataset, resized. Best regulariser: *red* TV, *green* ICTV, *blue* TGV$^{2}$; *top* SSIM, *middle* PSNR, *bottom* objective value

**Fig. 8:**
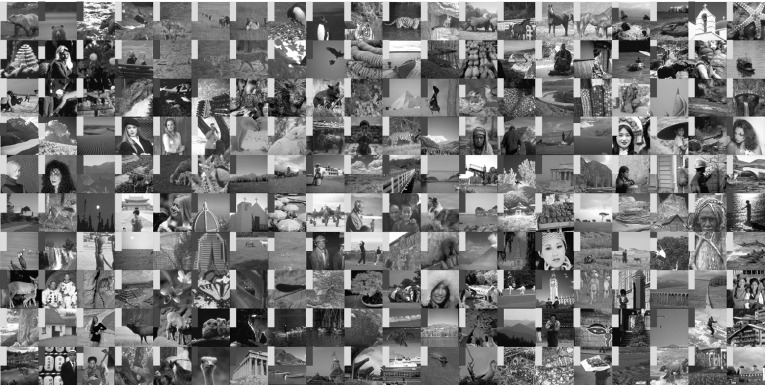
Ordering of regularisers with individual learning, $$L_2^2 $$ cost, and noise variance $$\sigma ^{2}=2$$, on the 200 images of the BSDS300 dataset, resized. Best regulariser: *red* TV, *green* ICTV, *blue* TGV$$^{2}$$; *top* SSIM, *middle* PSNR, *bottom* objective value

**Fig. 9:**
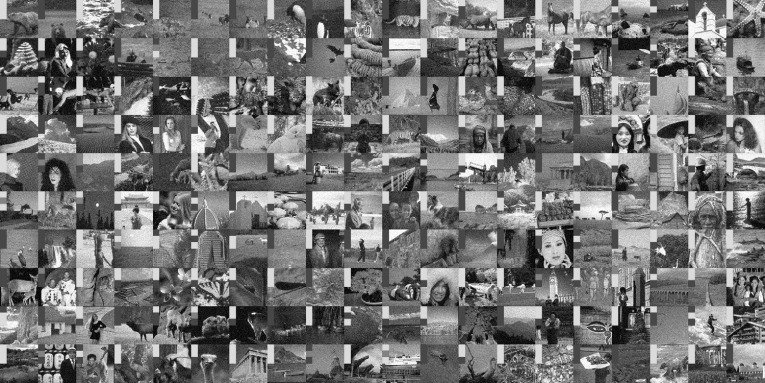
Ordering of regularisers with individual learning, $L_\eta ^1 \nabla$ cost, and noise variance $\sigma ^{2}=20$, on the 200 images of the BSDS300 dataset, resized. Best regulariser: *red* TV, *green* ICTV, *blue* TGV$^{2}$; *top* SSIM, *middle* PSNR, *bottom* objective value

**Fig. 10:**
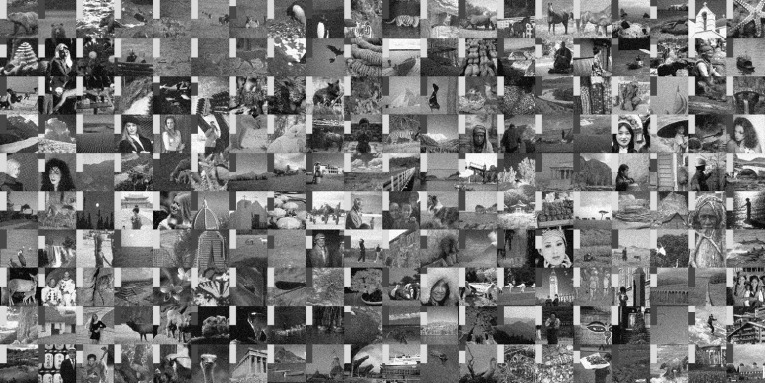
Ordering of regularisers with individual learning, $$L_2^2 $$ cost, and noise variance $$\sigma ^{2}=20$$, on the 200 images of the BSDS300 dataset, resized. Best regulariser: *red* TV, *green* ICTV, *blue* TGV$$^{2}$$; *top* SSIM, *middle* PSNR, *bottom* objective value

To obtain a statistically significant outlook to the performance of different regularisers and cost functionals, we made use of the Berkeley segmentation dataset BSDS300 [36], displayed in Fig. 6. We resized each image to 128 pixels on its shortest edge and take the $128\times 128$ top left square of the image. To this dataset, we applied pixelwise Gaussian noise of variance $\sigma ^2=2,10$, and 20. We tested the performance of both cost functionals, ${L_\eta ^1\!\nabla }$ and ${L_2^2}$, as well as the $\text {TGV}^2$, $\text {ICTV}$, and $\text {TV}$ regularisers on this dataset, for all noise levels. In the first instance, reported in Figs. 7, 8, 9 and 10 (noise levels $\sigma ^2=2,20$ only), and Tables 3, 4 and 5, we applied the proposed bilevel learning model on each image individually, to learn the optimal parameters specifically for that image, and a corresponding noisy image for all of the noise levels separately. For the algorithm, we use the same parametrisation as presented in Sect. 5.1.

The figures display the noisy images and indicate by colour coding the best result as judged by the structural similarity measure SSIM [46], PSNR and the objective function value (${L_\eta ^1\!\nabla }$ or ${L_2^2}$ cost). These criteria are, respectively, the top, middle and bottom rows of colour-coding squares. Red square indicates that $\text {TV}$ performed the best, green square indicates that $\text {ICTV}$ performed the best and blue square indicates that $\text {TGV}^2$ performed the best—this is naturally for the optimal parameters for the corresponding regulariser and cost functional discovered by our algorithms.

In the tables, we report the information in a more concise numerical fashion, indicating the mean, standard deviation and median for all the different criteria (SSIM, PSNR and cost functional value), as well as the number of images for which each regulariser performed the best. We recall that SSIM is normalised to [0, 1], with higher value better. Moreover, we perform a statistical 95 paired t-test on each of the criteria, and a pair of regularisers, to see whether any pair of regularisers can be ordered. If so, this is indicated in the last row of each of the tables.

Overall, studying the t-test and other data, the ordering of the regularisers appears to be

$$\begin{aligned} \text {ICTV}> \text {TGV}^2 > \text {TV}. \end{aligned}$$

This is rather surprising, as in many specific examples, $\text {TGV}^2$ has been observed to perform better than $\text {ICTV}$, see Figs. 4 and 5, as well as [1, 5]. Only when the noise is high, appears $\text {TGV}^2$ to come on par with $\text {ICTV}$ with the ${L_\eta ^1\!\nabla }$ cost functional in Fig. 9 and Table 5.

A more detailed study of the results in Figs. 7, 8, 9 and 10 seems to indicate that $\text {TGV}^2$ performs better than $\text {ICTV}$ when the image contains large smooth areas, but $\text {ICTV}$ generally seems to perform better for images with more complicated and varying contents. This observation agrees with the results in Figs. 4 and 5, as well as [1, 5], where the images are of the former type.

**Table 3:** Regulariser performance with individual learning, $$L_2^2 $$ and $$L_\eta ^1 \nabla $$ costs and noise variance $$\sigma ^{2} =$$ 2; BSDS300 dataset, resized

	SSIM				PSNR				Value			
	Mean	Std	Med	Best	Mean	Std	Med	Best	Mean	Std	Med	Best
Noisy data	0.978	0.015	0.981	0	41.56	0.86	41.95	0	2.9E$^{4}$	3.1E$^{2}$	2.9E$^{4}$	0
$L_\eta ^1 \nabla -$TV	0.988	0.005	0.989	1	42.57	1.10	42.46	5	2.4E$^{4}$	3.7E$^{3}$	2.5E$^{4}$	1
$L_\eta ^1 \nabla -$ICTV	0.989	0.005	0.990	141	42.74	1.16	42.62	143	2.3E$^{4}$	3.9E$^{3}$	2.4E$^{4}$	137
$L_\eta ^1 \nabla -$TGV$^{2}$	0.989	0.005	0.989	58	42.70	1.17	42.55	52	2.4E$^{4}$	4.0E$^{3}$	2.5E$^{4}$	62
95 % *t* test	$\hbox {ICTV}> \hbox {TGV}^{2} > \hbox {TV}$				$\hbox {ICTV}> \hbox {TGV}^{2} > \hbox {TV}$				$\hbox {ICTV}> \hbox {TGV}^{2} > \hbox {TV}$			
$L_2^2 -$TV	0.988	0.005	0.988	2	42.64	1.14	42.50	2	0.41	0.08	0.43	2
$L_2^2 -$ICTV	0.988	0.005	0.989	142	42.79	1.18	42.64	148	0.39	0.08	0.41	148
$L_2^2 -$TGV$^{2}$	0.988	0.005	0.989	56	42.76	1.19	42.58	50	0.40	0.08	0.42	50
95 % *t* test	$\hbox {ICTV}> \hbox {TGV}^{2} > \hbox {TV}$				$\hbox {ICTV}> \hbox {TGV}^{2} > \hbox {TV}$				$\hbox {ICTV}> \hbox {TGV}^{2} > \hbox {TV}$

**Table 4:** Regulariser performance with individual learning, $$L_2^2 $$ and $$L_\eta ^1 \nabla $$ costs and noise variance $$\sigma ^{2} =$$ 10; BSDS300 dataset, resized

	SSIM				PSNR				Value			
	Mean	Std	Med	Best	Mean	Std	Med	Best	Mean	Std	Med	Best
Noisy data	0.731	0.120	0.744	0	27.72	0.88	28.09	0	1.4E$^{5}$	2.5E$^{3}$	1.4E$^{5}$	0
$L_\eta ^1 \nabla -$TV	0.898	0.036	0.900	4	31.28	1.63	30.97	8	7.3E$^{4}$	2.2E$^{4}$	7.3E$^{4}$	1
$L_\eta ^1 \nabla -$ICTV	0.906	0.034	0.909	139	31.54	1.68	31.21	142	7.1E$^{4}$	2.2E$^{4}$	7.1E$^{4}$	121
$L_\eta ^1 \nabla -$TGV$^{2}$	0.905	0.035	0.907	57	31.47	1.72	31.10	50	7.1E$^{4}$	2.2E$^{4}$	7.1E$^{4}$	78
95 % *t* test	ICTV > TGV$^{2}>$ TV				ICTV > TGV$^{2}>$ TV				ICTV > TGV$^{2}>$ TV			
$L_2^2 -$TV	0.897	0.033	0.898	9	31.54	1.76	31.15	2	5.52	1.89	5.51	2
$L_2^2 -$ICTV	0.903	0.032	0.903	131	31.72	1.76	31.33	148	5.30	1.81	5.35	148
$L_2^2 -$TGV$^{2}$	0.902	0.033	0.903	60	31.67	1.80	31.28	50	5.38	1.87	5.39	50
95 % *t* test	ICTV > TGV$^{2}>$ TV				ICTV > TGV$^{2}>$ TV				ICTV > TGV$^{2}>$ TV

**Table 5:** Regulariser performance with individual learning, $$L_2^2 $$ and $$L_\eta ^1 \nabla $$ costs and noise variance $$\sigma ^{2} = 20$$; BSDS300 dataset, resized

	SSIM				PSNR				Value			
	Mean	Std	Med	Best	Mean	Std	Med	Best	Mean	Std	Med	Best
Noisy data	0.505	0.143	0.516	0	21.80	0.92	22.14	0	2.8E$^{5}$	7.9E$^{3}$	2.8E$^{5}$	0
$L_\eta ^1 \nabla -$TV	0.795	0.063	0.799	7	27.27	1.64	27.02	11	1.0E$^{5}$	3.5E$^{4}$	9.7E$^{4}$	1
$L_\eta ^1 \nabla -$ICTV	0.810	0.061	0.814	120	27.52	1.66	27.24	125	9.7E$^{4}$	3.4E$^{4}$	9.6E$^{4}$	79
$L_\eta ^1 \nabla -$TGV$^{2}$	0.808	0.062	0.814	73	27.50	1.74	27.15	64	9.8E$^{4}$	3.5E$^{4}$	9.5E$^{4}$	120
95 % *t* test	ICTV > TGV$^{2}>$ TV				ICTV, TGV$^{2}>$ TV				ICTV, TGV$^{2}>$ TV			
$L_2^2 -$TV	0.802	0.056	0.804	8	27.70	1.93	27.28	0	13.65	5.53	13.14	0
$L_2^2 -$ICTV	0.811	0.056	0.816	126	27.86	1.91	27.45	138	13.14	5.22	12.62	138
$L_2^2 -$TGV$^{2}$	0.810	0.057	0.814	66	27.83	1.94	27.41	62	13.28	5.38	12.77	62
95 % *t* test	ICTV > TGV$^{2}>$ TV				ICTV > TGV$^{2}>$ TV				ICTV > TGV$^{2}>$ TV			

One possible reason for the better performance of $\text {ICTV}$ could be that $\text {TGV}^2$ has more degrees of freedom—in $\text {ICTV}$ we essentially constrain $w=\nabla v$ for some function *v*—and therefore overfits to the noisy data, until the noise level becomes so high that overfitting would become too high for any parameter. To see whether this is true, we also performed batch learning, learning a single set of parameters for all images with the same noise level. That is, we studied the model

$$\begin{aligned}&\min _{\vec \alpha } \sum _{i=1}^N F_i(u_{i,\vec \alpha }) \quad \text {s.t.}\quad u_{i,\vec \alpha } \in \mathop {{{\mathrm{arg\,min}}}}\limits _{u\in H^1(\Omega )} \frac{1}{2}\Vert f_i-u\Vert _{L^2(\Omega )}^2\\&+\, R_{\vec \alpha }^{\gamma ,\mu }(u), \end{aligned}$$

with

$$\begin{aligned} F_i(u)= & {} \frac{1}{2}\Vert f_{0,i}-u\Vert ^2_{L^2(\Omega )}, \quad \text {or}\quad F_i(u)\\= & {} \int _\Omega |\nabla (f_{0,i}-u)|_\gamma \,dx, \end{aligned}$$

where $\vec \alpha =(\alpha , \beta )$, $f_1,\ldots ,f_N$ are the $N=200$ noisy images with the same noise level, and $f_{0,1},\ldots ,f_{0,N}$ the original noise-free images.

**Fig. 11:**
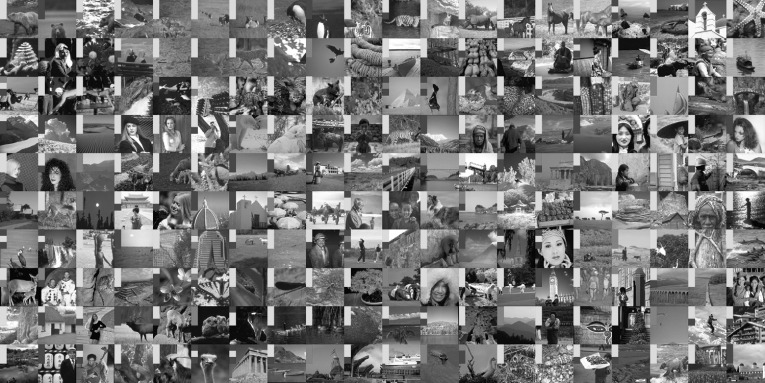
Ordering of regularisers with batch learning, $L_\eta ^1 \nabla$ cost, and noise variance $\sigma ^{2}=2$, on the 200 images of the BSDS300 dataset, resized. Best regulariser: *red* TV, *green* ICTV, *blue* TGV$^{2}$; *top* SSIM, *middle* PSNR, *bottom* objective value

**Fig. 12:**
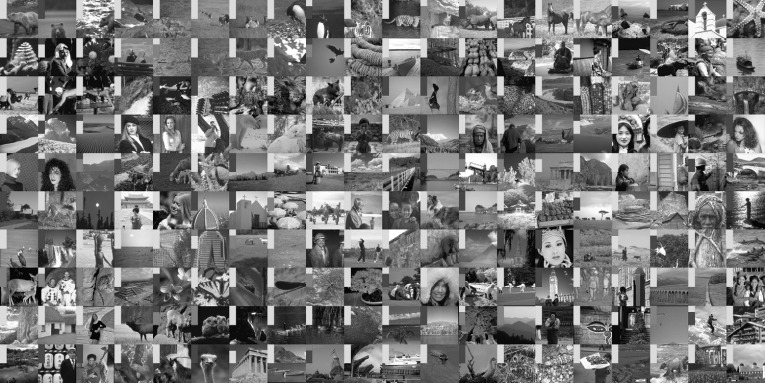
Ordering of regularisers with batch learning, $$L_2^2 $$ cost, and noise variance $$\sigma ^{2}=2$$, on the 200 images of the BSDS300 dataset, resized. Best regulariser: *red* TV, *green* ICTV, *blue* TGV$$^{2}$$; *top* SSIM, *middle* PSNR, *bottom* objective value

**Fig. 13:**
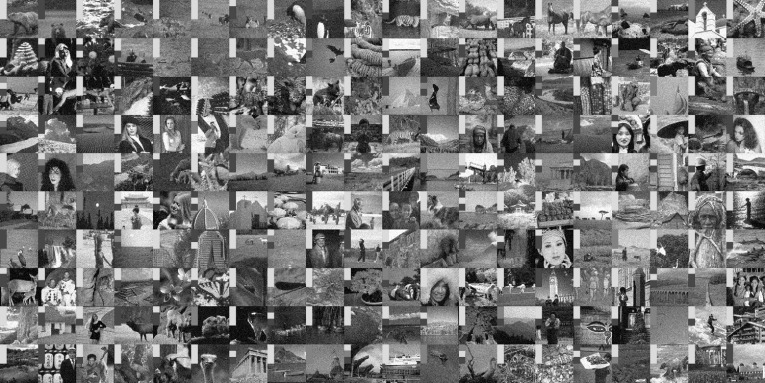
Ordering of regularisers with batch learning, $L_\eta ^1 \nabla$ cost, and noise variance $\sigma ^{2}=20$, on the 200 images of the BSDS300 dataset, resized. Best regulariser: *red* TV, *green* ICTV, *blue* TGV$^{2}$; *top* SSIM, *middle* PSNR, *bottom* objective value

**Fig. 14:**
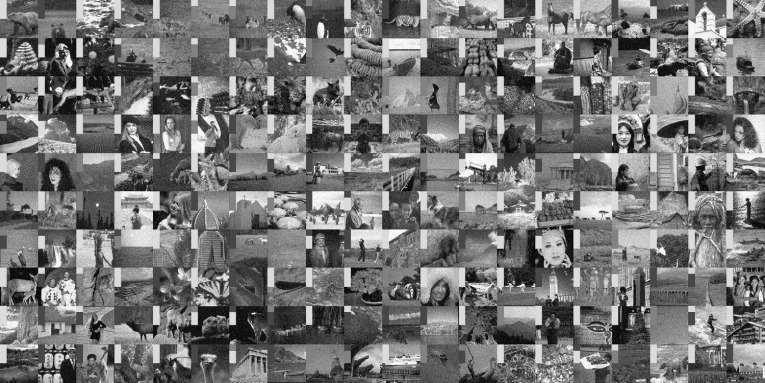
Ordering of regularisers with batch learning, $$L_2^2 $$, cost, and noise variance $$\sigma ^{2}=20$$, on the 200 images of the BSDS300 dataset, resized. Best regulariser: *red* TV, *green* ICTV, *blue* TGV$$^{2}$$; *top* SSIM, *middle* PSNR, *bottom* objective value

The results are shown in Figs. 11, 12, 13 and 14 (noise levels $\sigma ^2=2,20$ only), and Tables 6, 7 and 8. The results are still roughly the same as with individual learning. Again, only with high noise in Table 8, $\text {TGV}^2$ does not lose to $\text {ICTV}$. Another interesting observation is that $\text {TV}$ starts to be frequently the best regulariser for individual images, although still statistically does worse than either $\text {ICTV}$ or $\text {TGV}^2$.

**Fig. 15:**
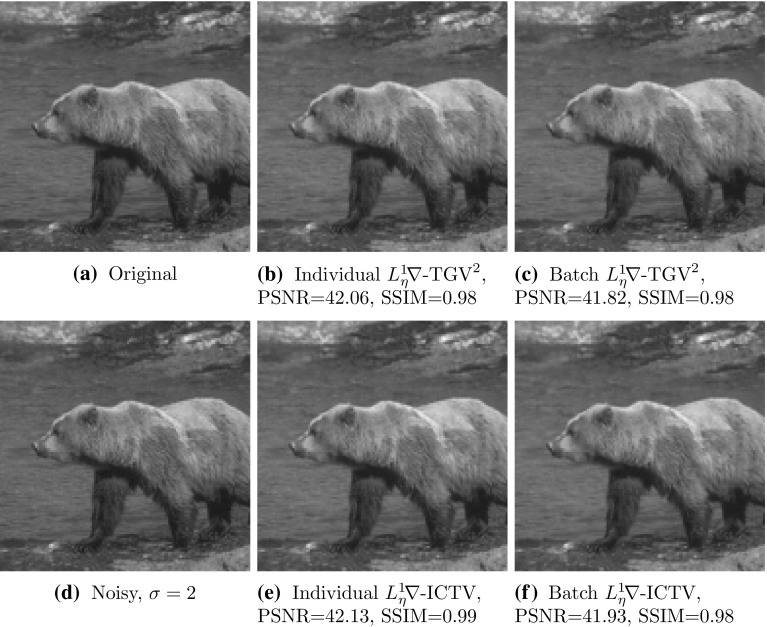
Image for which $\text {ICTV}$ performs better than $\text {TGV}^2$, $\sigma =2$

**Fig. 16:**
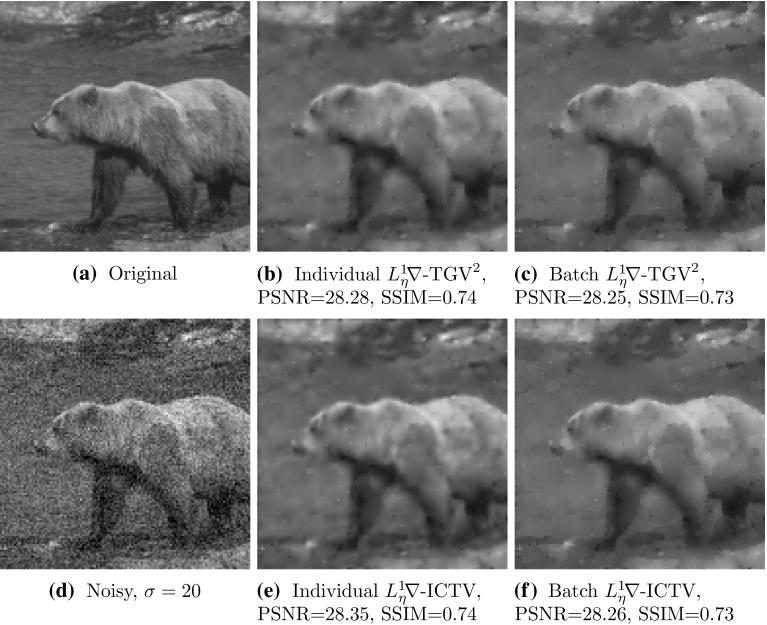
Image for which $\text {ICTV}$ performs better than $\text {TGV}^2$, $\sigma =20$

**Fig. 17:**
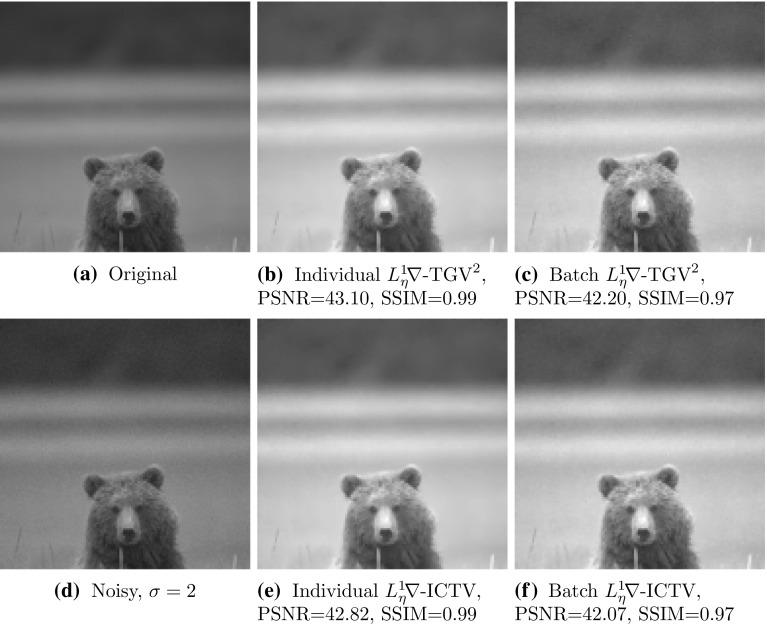
Image for which $\text {TGV}^2$ performs better than $\text {ICTV}$, $\sigma =2$

**Fig. 18:**
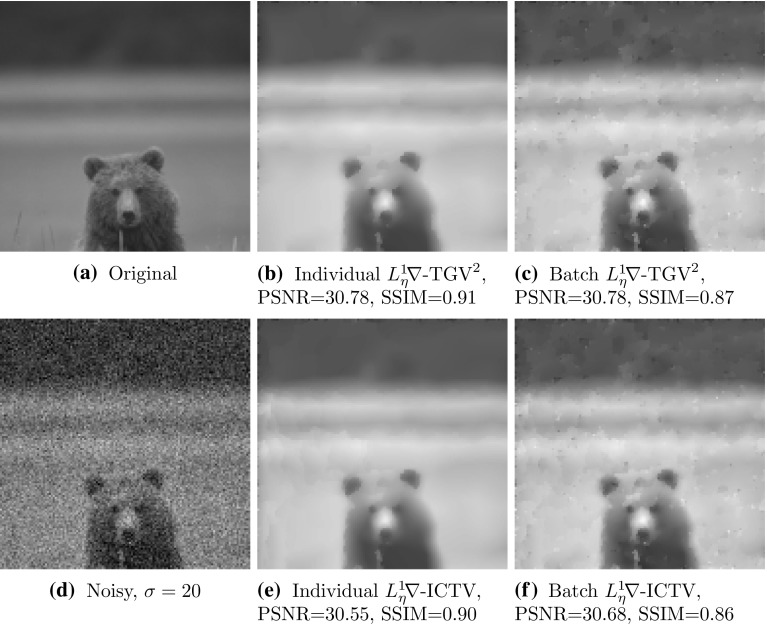
Image for which $\text {TGV}^2$ performs better than $\text {ICTV}$, $\sigma =20$

For the first image of the dataset, $\text {ICTV}$ does in all of the Figs. 7, 8, 9, 10, 11, 12, 13 and 14 better than $\text {TGV}^2$, while for the second image, the situation is reversed. We have highlighted these two images for the ${L_\eta ^1\!\nabla }$ cost in Figs. 15, 16, 17 and 18, for both noise levels $\sigma =2$ and $\sigma =20$. In the case where $\text {ICTV}$ does better, hardly any difference can be observed by the eye, while for second image, $\text {TGV}^2$ clearly has less staircasing in the smooth areas of the image, especially with the noise level $\sigma =20$.

Based on this study, it therefore seems that $\text {ICTV}$ is the most reliable regulariser of the ones tested, when the type of image being processed is unknown, and low SSIM, PSNR or ${L_\eta ^1\!\nabla }$ cost functional value is desired. But as can be observed for individual images, it can within large smooth areas exhibit artefacts that are avoided by the use of $\text {TGV}^2$.

### The Choice of Cost Functional

The ${L_2^2}$ cost functional naturally obtains better PSNR than ${L_\eta ^1\!\nabla }$, as the two former are equivalent. Comparing the results for the two cost funtionals in Tables 3, 4 and 5, we may however observe that for low noise levels $\sigma ^2=2,10$, and generally for batch learning, ${L_\eta ^1\!\nabla }$ attains better (higher) SSIM. Since SSIM better captures [46] the visual quality of images than PSNR, this recommends the use of our novel total variation cost functional ${L_\eta ^1\!\nabla }$. Of course, one might attempt to optimise the SSIM. This is however a non-convex functional, which will pose additional numerical challenges avoided by the convex total variation cost.

**Table 6:** Regulariser performance with batch learning, $$L_\eta ^1 \nabla $$ and $$L_2^2 $$ costs, noise variance $$\sigma ^{2} =$$ 2; BSDS300 dataset, resized

	SSIM				PSNR				Value			
	Mean	Std	Med	Best	Mean	Std	Med	Best	Mean	Std	Med	Best
Noisy data	0.978	0.015	0.981	16	41.56	0.86	41.95	24	2.9E$^{4}$	3.1E$^{2}$	2.9E$^{4}$	16
$L_\eta ^1 \nabla -$TV	0.987	0.006	0.988	23	42.43	1.07	42.37	21	2.5E$^{4}$	3.4E$^{3}$	2.5E$^{4}$	20
$L_\eta ^1 \nabla -$ICTV	0.988	0.006	0.989	119	42.56	1.06	42.51	135	2.4E$^{4}$	3.5E$^{3}$	2.5E$^{4}$	113
$L_\eta ^1 \nabla -$TGV$^{2}$	0.987	0.006	0.989	42	42.51	1.09	42.44	20	2.4E$^{4}$	3.6E$^{3}$	2.5E$^{4}$	51
95 % *t* test	ICTV > TGV$^{2}>$ TV				ICTV > TGV$^{2}>$ TV				ICTV > TGV$^{2}>$ TV			
$L_2^2 -$TV	0.986	0.007	0.987	13	42.46	0.95	42.43	17	0.42	0.07	0.43	17
$L_2^2 -$ICTV	0.987	0.007	0.988	139	42.57	0.95	42.56	128	0.41	0.07	0.42	128
$L_2^2 -$TGV$^{2}$	0.987	0.007	0.988	38	42.53	0.97	42.51	40	0.41	0.07	0.42	40
95 % *t* test	ICTV > TGV$^{2}>$ TV				ICTV > TGV$^{2}>$ TV				ICTV > TGV$^{2}>$ TV

**Table 7:** Regulariser performance with batch learning, $$L_\eta ^1 \nabla $$ and $$L_2^2 $$ costs, noise variance $$\sigma ^{2}=$$ 10; BSDS300 dataset, resized

	SSIM				PSNR				Value			
	Mean	Std	Med	Best	Mean	Std	Med	Best	Mean	Std	Med	Best
Noisy data	0.731	0.120	0.744	8	27.72	0.88	28.09	2	1.4E$^{5}$	2.5E$^{3}$	1.4E$^{5}$	0
$L_\eta ^1 \nabla -$TV	0.893	0.035	0.897	23	31.24	1.87	30.94	23	7.5E$^{4}$	2.2E$^{4}$	7.3E$^{4}$	18
$L_\eta ^1 \nabla -$ICTV	0.897	0.034	0.902	134	31.36	1.81	31.11	150	7.4E$^{4}$	2.2E$^{4}$	7.2E$^{4}$	107
$L_\eta ^1 \nabla -$TGV$^{2}$	0.896	0.035	0.901	35	31.31	1.88	31.01	25	7.4E$^{4}$	2.3E$^{4}$	7.2E$^{4}$	75
95 % *t* test	ICTV > TGV$^{2}>$ TV				ICTV > TGV$^{2}>$ TV				ICTV, TGV$^{2}>$ TV			
$L_2^2 -$TV	0.887	0.035	0.889	29	31.31	1.50	31.15	25	5.72	1.91	5.51	25
$L_2^2 -$ICTV	0.889	0.036	0.893	127	31.41	1.44	31.28	131	5.57	1.83	5.37	131
$L_2^2 -$TGV$^{2}$	0.888	0.035	0.891	44	31.38	1.50	31.20	44	5.64	1.90	5.44	44
95 % *t* test	ICTV > TGV$^{2}>$ TV				ICTV > TGV$^{2}>$ TV				ICTV > TGV$^{2}>$ TV

**Table 8:** Regulariser performance with batch learning, $$L_\eta ^1 \nabla $$ and $$L_2^2 $$ costs, noise variance $$\sigma ^{2} =$$ 20; BSDS300 dataset, resized

	SSIM				PSNR				Value			
	Mean	Std	Med	Best	Mean	Std	Med	Best	Mean	Std	Med	Best
Noisy data	0.505	0.143	0.516	4	21.80	0.92	22.14	1	2.8E$^{5}$	7.9E$^{3}$	2.8E$^{5}$	0
$L_\eta ^1 \nabla -$TV	0.789	0.067	0.798	18	27.37	2.13	26.98	24	1.0E$^{5}$	3.7E$^{4}$	9.8E$^{4}$	14
$L_\eta ^1 \nabla -$ICTV	0.795	0.065	0.804	139	27.46	2.10	27.05	141	1.0E$^{5}$	3.6E$^{4}$	9.6E$^{4}$	91
$L_\eta ^1 \nabla -$TGV$^{2}$	0.794	0.066	0.804	39	27.44	2.12	27.04	34	1.0E$^{5}$	3.7E$^{4}$	9.6E$^{4}$	95
95 % *t* test	ICTV > TGV$^{2}>$ TV				ICTV > TGV$^{2}>$ TV				TGV$^{2}>$ ICTV > TV			
$L_2^2 -$TV	0.786	0.053	0.790	31	27.50	1.71	27.27	33	14.11	5.78	13.16	33
$L_2^2 -$ICTV	0.790	0.054	0.790	123	27.56	1.64	27.37	119	13.84	5.54	12.75	119
$L_2^2 -$TGV$^{2}$	0.789	0.053	0.793	46	27.55	1.70	27.33	48	13.93	5.73	12.95	48
95 % *t* test	ICTV, TGV$^{2}>$ TV				ICTV, TGV$^{2}>$ TV				ICTV > TGV$^{2}>$ TV			

## Conclusion and Outlook

In this paper, we propose a bilevel optimisation approach in function space for learning the optimal choice of parameters in higher-order total variation regularisation. We present a rigorous analysis of this optimisation problem as well as a numerical discussion in the context of image denoising.

Analytically, we obtain the existence results for the bilevel optimisation problem and prove the Fréchet differentiability of the solution operator. This leads to the existence of Lagrange multipliers and a first-order optimality system characterising optimal solutions. In particular, the existence of an adjoint state allows to obtain a cost functional gradient formula which is of importance in the design of efficient solution algorithms.

We make use of the bilevel learning approach, and the theoretical findings, to compare the performance—in terms of returned image quality—of TV, ICTV and TGV regularisation. A statistical analysis, carried out on a dataset of 200 images, suggests that ICTV performs slightly better than TGV, and both perform better than TV, in average. For denoising of images with a high noise level, ICTV and TGV score comparably well. For images with large smooth areas, TGV performs better than ICTV.

Moreover, we propose a new cost functional for the bilevel learning problem, which exhibits interesting theoretical properties and has a better behaviour with respect to the PSNR related L$^2$ cost used previously in the literature. This study raises the question of other, alternative cost functionals. For instance, one could be tempted to used the SSIM as cost, but its non-convexity might present several analytical and numerical difficulties. The new cost functional, proposed in this paper, turns out to be a good compromise between image quality measure and analytically tractable cost term.
